# Total Synthesis of Flocoumafen via Knoevenagel Condensation and Intramolecular Ring Cyclization: General Access to Natural Product

**DOI:** 10.3390/molecules17022091

**Published:** 2012-02-21

**Authors:** Jae-Chul Jung, Eunyoung Lim, Yongnam Lee, Dongguk Min, Jeremy Ricci, Oee-Sook Park, Mankil Jung

**Affiliations:** 1 Department of Neuroscience and Medical Research Institute, School of Medicine, Ewha Womans University, Seoul 158-710, Korea; 2 Department of Chemistry, Yonsei University, Seoul 120-749, Korea; 3 Department of Chemistry, Institute for Basic Sciences, College of Natural Sciences, Chungbuk National University, Cheongju 361-763, Chungbuk, Korea

**Keywords:** flocoumafen, Knoevenagel condensation, tetralone, 2D NMR analysis, computer simulation

## Abstract

The total synthesis and structure determination of *cis-* and *trans*-flocoumafen was described. The key synthetic steps involve Knoevenagel condensation with *p*-methoxybenzaldehyde, *in situ* decarboxylation and intramolecular ring cyclization to construct the tetralone skeleton. Stereospecific reduction of the *O*-alkylated ketone **13** afforded good yield of precusor alcohol **5**. Final coupling of alcohol **5** with 4-hydroxy-coumarin yielded flocoumafen (**1**). Separation and structure determination of *cis*- and *trans*-flocoumafen through 2D NMR analyses-assisted computer simulation techniques for the evaluation of anticoagulant activities are reported for the first time. This method is useful for generating the core tetralone skeleton of 4-hydroxycoumarin derivatives and provides a generalized access to various warfarin type anticoagulants.

## 1. Introduction

**Figure 1:**
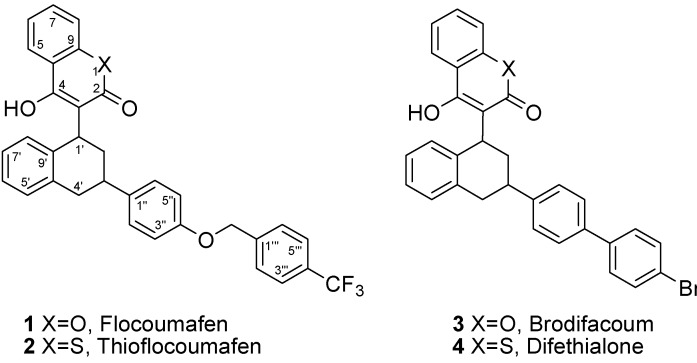
Structures of warfarin type anticoagulants **1**–**4**.

The physiological potential of naturally occurring coumarins has attracted considerable attention. In particular, 4-hydroxycoumarin anticoagulant agents, widely used as rodenticides, are of interest for cell growth stimulation, bacteriostatic activity, and the treatment of thrombotic diseases [1]. Many of these compounds have side effects, including the warfarin-related “purple toes” syndrome and inhibition of vitamin K epoxide reductase [2]. Currently, warfarin type anticoagulants such as brodifacoum [3], bromadiolone [4], flocoumafen [5], difenacoum [6], thioflocoumafen [7], and difethilone [8] (Figure 1) have been developed to control rodents with low toxicity [9] and can also be used in low concentrations for the treatment of human circulatory diseases [10]. Although warfarin has been tentatively used to control rodents, it is a direct hazard to domestic animals and wild mammals. Flocoumafen (FCF, **1**) is a potent, effective anticoagulant agent in the 4-hydroxycoumarin class.

A recent report described the development of a biological screening assay to detect anticoagulant rodenticides based on inhibitory action on the vitamin K epoxide reductase protein complex [11]. Previous research has addressed synthetic approaches to warfarin type anticoagulants through the formation of the carbon backbone using organocopper methodology, ring cyclization, and coupling with a 4-hydroxycoumarin moiety [12]. The development of new methods for the efficient and selective preparation of flocoumafen is of great interest in organic and medicinal chemistry due to the frequent occurrence of this structural class in biologically active compounds and as valuable synthetic intermediates for potential new pharmaceuticals.

In preliminary communications, we developed a method for multi-step synthesis of 4-hydroxycoumarin derivatives using Friedel-Crafts, Refortmasky, and ring cyclization and reported the biological activities of various derivatives [13]. As part of our continuing interest in the synthesis of 4-hydroxycoumarin derivatives for potential use as anticoagulants, we wanted to establish a practical and efficient synthesis of flocoumafen. In this report, we describe the efficient total synthesis of flocoumafen starting from readily available 4-methoxybenzaldehyde by Knoevenagel condensation, Michael 1,4-addition reaction, ring cyclization, and a final coupling reaction. In addition, separation and structure determination of *cis*- and *trans*-flocoumafen through 2D NMR analyses and computer simulation techniques are reported for the evaluation of anticoagulant activities.

## 2. Results and Discussion

### 2.1. Total Synthesis

**Scheme 1:**
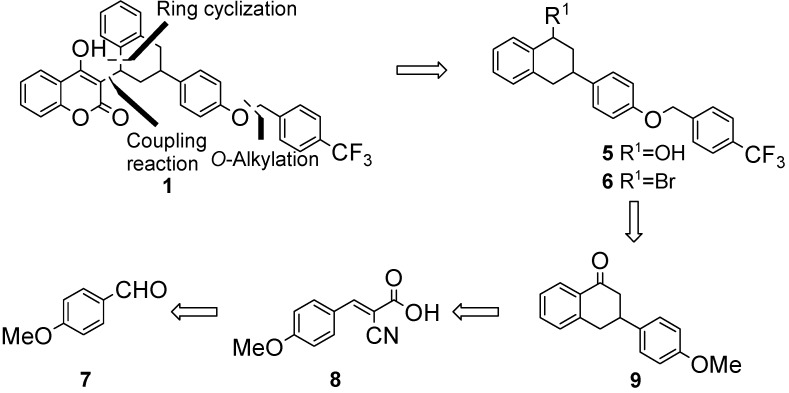
Retrosynthetic analysis of flocoumafen (**1**).

As shown in Scheme 1, this synthetic method is practical and provides generalized access to the target flocoumafen and various other warfarin type anticoagulants. The retrosynthetic analysis shown in Scheme 1 provides a strategy for easy access to flocoumafen (**1**) through a reaction sequence involving Knoevenagel condensation of *p*-methoxybenzaldehyde (**7**) with ethyl cyanoacetate, a Michael 1,4-addition, and construction of the tetralone skeleton **9** via intramolecular ring cyclization [5], and preparation of alcohol **5** by stereospecific reduction of ketone **9**.

**Scheme 2:**
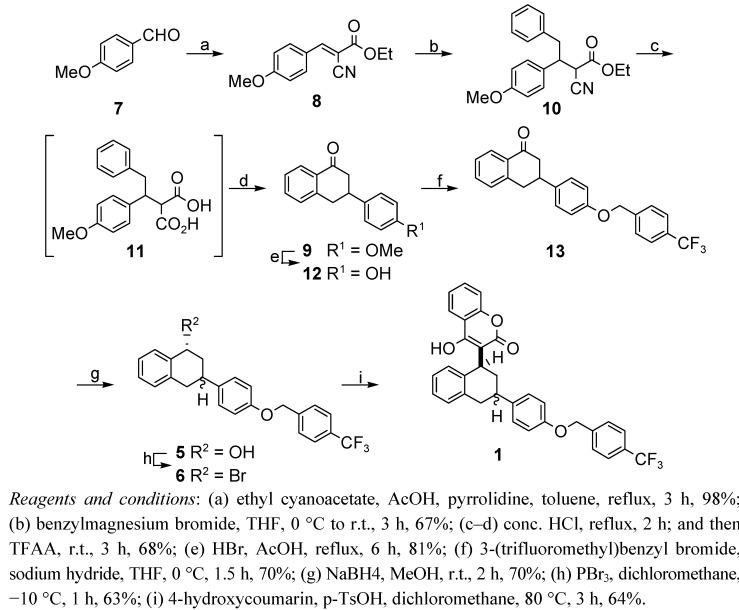
Total synthesis of flocoumafen (**1**).

Analogous to the known Knoevenagel condensation [14,15,16,17,18], we found that the reaction of ethyl cyanoacetate with *p*-methoxybenzaldehyde (**7**) in the presence of acetic acid and pyrrolidine generated an excellent yield (98%) of the desired product **8** in Scheme 2. This result suggests that ethyl cyanoacetate has better reactivity due to its relatively high α-proton acidity.

Having successfully investigated Knoevenagel condensation conditions to yield ketoester **8** in excellent yield, we then examined approaches to generate the key synthetic intermediate, tetralone **9**. Compound **8** was treated with freshly prepared benzylmagnesium bromide in dry THF to afford **10** in 67% yield, which was readily oxidized in acidic media to produce diacid **11**. *In situ* decarboxylation of **11** was accomplished with hydrochloric acid to generate the monoacid, which then underwent an intramolecular ring cyclization using trifluoroacetic anhydride (TFAA) to give tetralone **9** in overall 68% yield. Demethylation of the methoxy group of compound **9** was accomplished with hydrobromic acid in acetic acid to generate phenol **12**, which was treated with freshly prepared 3-(trifluoromethyl)benzyl bromide in the presence of sodium hydride in THF at 0 °C to give the *O*-alkylated product **13** in 70% yield over two steps. Ketone **13** was treated with sodium borohydride in MeOH to afford the secondary alcohol **5**, which was readily transformed into bromide **6** using phosphorus tribromide in dichloromethane in 44% yield over two steps. At this stage, we noticed that reduction of the ketone of compound **13** with sodium borohydride in methanol exclusively produced *cis* alcohol **5**. This result was somewhat surprising, since no particular steric hindrance to approach of the reducing species from either carbonyl face would be anticipated from molecular models. A reasonable explanation for this stereochemical anomaly was included in a preliminary communication [13].

To complete the synthesis, coupling reactions of either alcohol **5** or bromide **6** with 4-hydroxycoumarin were attempted under acidic conditions. Alcohol **5** was treated with *p*-toluenesulfonic acid to give flocoumafen (**1**) in 64% yield as a 1:1 structurally isomeric mixture of *cis*-flocoumafen (*cis*-FCF) and *trans*-flocoumafen (*trans*-FCF). On the other hand, the coupling reaction of bromide **6** with 4-hydroxy-coumarin was not effective for the preparation of flocoumafen (**1**) due to dehydrohalogenation. Synthetic flocoumafen consists of a mixture of *cis* and *trans*, and its separation for three- dimensional structure determinations has never been reported to date in the literature. For the purpose of the evaluation of anticoagulant activities, we separated the diastereomers into *cis*-FCF and *trans*-FCF using flash silica gel column chromatography. In addition, ethyl acetate could be used as a recrystallization solvent to exclusively afford the *cis*-form of flocoumarin (**1**) with 99% purity, while a solvent mixture of ethyl acetate/hexane (1:4, v/v) gave predominantly the *trans*-form with 98% purity.

The anticoagulant activities of the individual isomers along with the mixture of flocoumafen will be reported in due course. 

### 2.2. Structure Determination

The structures of *cis*- and *trans*-flocoumafen were characterized through 2D NMR analyses and further computer simulation techniques. High-resolution 1D and 2D NMR analyses of *cis*-flocoumafen allowed us to assign most of the proton and carbon peaks. A homo-correlation spectroscopy (COSY) experiment was useful in clarifying the regioselective assignment of aliphatic and aromatic protons. The proton at the 1' position was coupled with H2' and H3". The benzylic proton was coupled with H2" and H6". The ^13^C-NMR assignments of protonated carbons were established using the heteronuclear multiple quantum coherence technique (HMQC). The C3' signal of *cis*-flocoumafen had a chemical shift of *δ* = 39.8, while the C3' signal of *trans*-flocoumafen was assigned further upfield at *δ* = 36.5. However, the C4' signal of *cis*-flocoumafen was further upfield, *δ* = 38.6, than was the C4' signal of *trans*-flocoumafen which was assigned as *δ* = 39.8. These results indicate that the C3' of *cis*-flocoumafen has a relatively smaller bond angle than does the C3' of *trans*-flocoumafen. Interestingly, the benzylic secondary carbons of both *cis*-and *trans*-flocoumafen had the same chemical shift at *δ* = 69.3 (Table 1 and Table 2).

**Table 1:** ^1^H-NMR (500 MHz) and ^13^C-NMR (125 MHz) for *cis*-flocoumafen in CDCl_3_.

Position	^1^H (multi *^a^*, *J* in Hz)	^13^C *^b^*	HMBC *^c^*	NOESY *^d^*
5	7.72 (d, 7.5)	123.1	4, 7, 9, CH	H6^s^, H7^m^
1'	4.87 (dd,5.5, 5.5)	37.5		H2'^m^, H3'^w^
2'	2.52–2.42 (m)1.95–1.80 (m)	36.7	3', 4', CH_2_	H1'^m^, H3'^m^, H2"^w^, H6"^w^
3'	3.13–3.02 (m)	9.8	2", 6", 4', 10', CH	H2"^s^, H6"^s^, H2'^m^, H1'^w^
4'	3.13–3.02 (m)	38.6	5', 3', CH_2_	H2'^s^, H1'^w^, H2"^w^, H6"^w^
2"	7.21 (d, 9.0)	127.9		H3"^s^
6"	7.21 (d, 9.0)	127.9		H5"^s^

*^a^* Multi., multiplicity: s, singlet; d, doublet; t, triplet; q; quartet; dd, doublet of doublet; m, multiplet. *^b^* The chemical shifts were extracted from ^13^C and HMQC experiments. *^c^* The correlations were assigned as quaternary, tertiary and secondary carbons from HMBC and DEPT (135) analysis. *^d^* NOESY intensities are marked as strong (s), medium (m), and weak (w).

**Table 2:** ^1^H-NMR (500 MHz) and ^13^C-NMR (125 MHz) for *trans*-flocoumafen in CDCl_3_.

Position	^1^H (multi *^a^*, *J* in Hz)	^13^C *^b^*	HMBC *^c^*	NOESY *^d^*
5	7.66 (dd, 1.5, 1.5)	123.9	7, CH	H6^s^, H7^m^
1'	4.72 (t, 4.0)	37.5	3', CH	H2'^m^, H8'^w^
2'	2.36-2.32 (m)	35.9	4', CH_2_	H1'^m^, H3'^m^, H4'^w^
3'	3.12-2.99 (m)	36.5	2", 6", 4', CH	H2"^s^, H6"^s^, H2'^m^, H1'^w^
4'	3.23 (d, 12.0)	39.8	1", 3', 5', 9', CH_2_	H2'^s^, H5'^m^, H2"^m^, H6"^m^, H1'^w^
2"	7.16 (d, 8.5)	128.0		H3"^s^, H2'^w^
6"	7.16 (d, 8.5)	128.0		H5"^s^, H2'^w^

Superscript letters (a–d) in Table 2 are identical to those of Table 1.

The assignments of the protons on H5, H2', and H3' were confirmed by the hetero multiple bound correlation technique (HMBC) of C4, C7, C9; C3', C4'; and C4', C2", C6", respectively. The benzylic protons showed couplings to C1'" and C4", and the benzyl carbon was correlated with the H2'" and H6'" protons. The HMBC experiment also enabled us to corroborate the presence of the trifluoro methyl group at the C4'" position.

The Nuclear Overhauser Enhancement spectroscopy (NOESY) spectrum showed a long-range correlation between H1' and H3' confirming the stereochemistry of *cis*- and *trans*-flocoumafen. The H1' protons of *cis*-flocoumafen were coupled with H2' and H3', while the H1' protons of *trans*-flocoumafen had a coupling only with the H2' proton and did not display coupling with H3', as shown in Table 1. These results indicate that the H1' benzylic proton of the *cis*-isomer causes a substantial NOE enhancement of the C3' benzylic signal at 39.8. However, the *trans*-isomer had no detectable NOE effect upon irradiation of the H1' and H3' benzylic protons (Table 2). 

**Figure 2:**
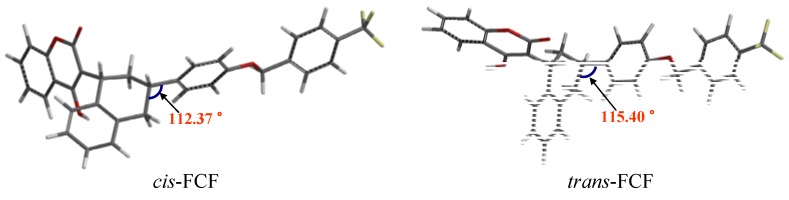
3D-structures of *cis*-flocoumafen (left) and *trans*-flocoumafen (right) generated using SPARTAN 06 for Windows.

We turned to computer simulations to help explain the structural differences between *cis*- and *trans*- flocoumafen (FCF). The structures of *cis*-FCF and *trans*-FCF shown in Figure 2 were geometrically optimized at the B3LYP/6-31G^**^ level [19] with the use of SPARTAN 06 for Windows [20] (see Supporting Information). The results of analysis of the calculated structures of the two isomers are revealing. Averaged values of bond lengths, bond angles and dihedral torsion angles for the structures (Table 3) were obtained.

**Table 3:** Geometrical parameters determined for *cis*- and *tran*s-flocoumafen using the B3LYP 6-31G^**^ basis set.

Parameter	*cis*-FCF	*trans*-FCF
	Bond lengths (Å)	
H1'-H2'	2.37	2.39
H1'-H3'	2.60	3.83
H5-C4	2.74	2.80
H5-C7	3.41	3.39
H5-C9	3.39	3.41
	Bond angle (deg)	
C4'-C3'-C1"	112.37	115.40
	Dihedral angles (deg)	
H5-C5-C6-C4	0.05	1.93
H5-C5-C8-C7	179.83	178.18
H5-C5-C6-C9	0.17	2.72
H2'-C2'-C3'-C4'	4.28	2.45

The C4'- C3'- C1'' bond angle of the *cis*-isomer is approximately 3° smaller than that of the *trans*-isomer (Figure 2 and Table 3), in good agreement with the results from the HMQC analysis. Moreover, the H1'- H2' and H1'- H3' bond distances in the *cis*-isomer, 2.37 Å and 2.60 Å, were notably shorter than in the *trans*-isomer, 2.39 Å and 3.83 Å, respectively. These results are verified by the calculated values from the B3LYP analysis for the NOE effect (see Table 3). Finally, we investigated the dihedral angles for the key differing bonds in the *cis* and *trans* isomers. These dihedral angles (H5-C5-C6-C4, H5-C5-C8-C7, H5-C5-C6-C9, H2'-C2'-C3'-C4') are shown in Table 2. As shown in Table 3, experimental values obtained from the NMR studies (Table 1 and (Table 2) could be used to satisfactorily explain the conformations of both the *cis-* and *trans-*flucoumafen (**1**) isomers (Figure 2).

## 3. Experimental

### 3.1. General

All commercial reagents and solvents were used as received without further purification unless specified [21]. Reaction solvents were distilled from calcium hydride for dichloromethane and from sodium metal and benzophenone for tetrahydrofuran. The reactions were monitored and the *R_f_* values determined using analytical thin layer chromatography (TLC) with Merck silica gel 60 and F-254 precoated plates (0.25-mm thickness). Spots on the TLC plates were visualized using ultraviolet light (254 nm) and a basic potassium permanganate solution or cerium sulfate/ammonium dimolybdate/sulfuric acid solution followed by heating on a hot plate. Flash column chromatography was performed with Merck silica gel 60 (230–400 mesh). ^1^H-NMR spectra were recorded on Bruker DPX-250, 400 or Varian Unity-Inova 500 Spectrometers. Proton chemical shifts are reported in ppm (δ) relative to internal tetramethylsilane (TMS, δ 0.00) or with the solvent reference relative to TMS employed as the internal standard (CDCl_3_, δ 7.26 ppm; d_4_-CD_3_OD, δ 3.31 ppm, d_6_-DMSO, δ 2.50 ppm). Data are reported as follows: chemical shift {multiplicity [singlet (s), doublet (d), triplet (t), quartet (q), and multiplet (m)], coupling constants [Hz], integration}. ^13^C-NMR spectra were recorded on Bruker DPX-250 (63 MHz), 400 (100 MHz) or Varian Unity-Inova 500 (125 MHz) spectrometers with complete proton decoupling. Carbon chemical shifts are reported in ppm (δ) relative to TMS with the respective solvent resonance as the internal standard (CDCl_3_, δ 77.0 ppm; d_4_-CD_3_OD, δ 49.0 ppm, d_6_-DMSO, δ 39.5 ppm). Infrared (IR) spectra were recorded on a Nicolet Model Impact FT-IR 400 spectrometer. Data are reported in wave numbers (cm^-1^). High resolution mass spectrometer (HRMS) analyses were recorded on an Applied Biosystems 4700 proteomics analyzer spectrometer.

### 3.2. General Procedure for the Knoevenagel Reaction: Synthesis of (E)-Ethyl 2-cyano-4-methoxy-cinnamte (**8**) [18]

To a solution of compound **7**, ethyl cyanoacetate (7.5 mmol) and acetic acid (2.2 mmol) in dry toluene (10 mL) pyrrolidine (0.7 mmol) was added dropwise at room temperature and the mixture was refluxed for 30 min under N_2_ gas. The reaction mixture was cooled to room temperature and then anisaldehyde (7.3 mmol) was added dropwise and refluxed for 3 h. The resulting mixture was cooled to room temperature and extracted by ethyl acetate (30 mL) and washed with water (20 mL) and brine (20 mL). The organic layer was dried over anhydrous MgSO_4_, filtered, and evaporated under reduced pressure. The crude was purified by flash column chromatography (silica gel, ethyl acetate/*n*-hexane 1:2, v/v) to give pure product **8** [14,15,16,17,18] in 98% yield. Yellow solid. mp 89–91 °C; R*_f_* = 0.6 (ethyl acetate/*n*-hexane = 1:2, v/v); IR (CHCl_3_, KBr) ν_max_ 3390, 2984, 2933, 2899, 2837, 2221, 1721, 1592, 1565, 1512, 1462, 1428, 1367, 1311, 1263, 1208, 1175, 1124, 1089, 1021, 835 cm^−1^; ^1^H-NMR (250 MHz, CDCl_3_) δ 8.13 (s, 1 H), 7.97 (d, 2 H, *J* = 8.9 Hz), 6.97 (d, 2 H, *J* = 8.9 Hz), 4.35 (q, 2 H, *J* = 7.1 Hz), 3.88 (s, 3 H), 1.38 (t, 3 H, *J* = 7.1 Hz); ^13^C-NMR (63 MHz, CDCl_3_) δ 163.7, 162.9, 133.5, 124.2, 116.1, 114.6, 99.2, 62.3, 55.5, 14.1; LC-MS (ESI+) *m/z* 254 [M+Na]. 

### 3.3. Synthesis of Compounds **10**, **9**, **12**, **13**, and **5**

*Ethyl 2-cyano-3-(4-methoxyphenyl)-4-phenylbutanoate* (**10**). To a solution of cinnamate **8d** (100 mg, 0.43 mmol) in dry THF (10 mL) was added to benzylmegansium bromide (0.52 mL, 0.52 mmol) under N_2_ gas at room temperature. The mixture was stirred for 3 h at same temperature and then the resulting mixture was extracted by ethyl acetate (20 mL) and washed with NaHCO_3_ (15 mL) and brine (15 mL). The organic layer was separated and dried over anhydrous MgSO_4_, filtered, and evaporated under reduced pressure. The crude was purified by flash column chromatography (silica gel, ethyl acetate/*n*-hexane = 1:2, v/v) to give pure product **10** (92 mg, 67% yield). Colorless oil. R*_f_* = 0.1 (*n*-hexane/acetone = 9:1, v/v); IR (CHCl_3_, KBr) ν_max_ 3462, 2932, 2838, 2331, 1732, 1611, 1584, 1515, 1496, 1455, 1384, 1369, 1303, 1252, 1207, 1179, 1114, 1030, 838 cm^−1^; ^1^H-NMR (400 MHz, CDCl_3_) δ 7.30–7.32 (m, 3H), 7.24–7.26 (m, 4H), 6.86–6.88 (d, 2H, *J* = 8.8 Hz), 4.02–4.07 (q, 3H, *J* = 6.8 Hz), 3.80 (s, 3H), 3.62 (s, 1H), 3.57–3.60 (m, 1H), 3.17 (d, 1H, *J* = 2.8 Hz), 3.15 (s, 1H), 1.09 (t, 3H, *J* = 7.2 Hz); ^13^C-NMR (100 MHz, CDCl_3_) δ 165.6, 159.4, 138.2, 130.5, 129.3, 129.2, 129.0, 128.5, 127.2, 126.8, 115.6, 114.2, 62.7, 55.4, 40.0, 38.9, 31.8, 14.3, 14.0; LC-MS (ESI+) *m/z* 346 [M+Na]. HRMS: *m/z* = 324.1612 (calcd. 324.1600 for C_2__0_H_22_NO_3_: [M+H]^+^).

#### 3.3.1. 3-(4-Methoxyphenyl)-l-tetralone (**9**)

Butanoate **10** (35.6 mg, 0.11 mmol) was added to conc. HCl (5 mL) and refluxed for 2 h. The mixture was extracted by dichloromethane (20 mL) and washed with water (15 mL). The organic layer was dried over MgSO_4_, filtered, and evaporated under reduced pressure. Then the crude **11** was added to trifluoroacetic anhydride (TFAA) (1 mL) at 0 °C. The mixture was stirred at room temperature for 3 h. The reaction mixture purified by flash column chromatography (silica gel, ethyl acetate/*n*-hexane = 1:2, v/v) to give pure product **9** (19 mg, 68%). White solid. mp 102–105 °C; R*_f_* = 0.3 (ethyl acetate/*n*-hexane = 1:2, v/v); IR (CHCl_3_, KBr) ν_max_ 1675 1505 1245 1025 cm^−1^; ^1^H-NMR (250 MHz, CDCl_3_) δ 7.90–8.30 (m, 1H), 7.07–7.73 (m, 5H), 6.83 (d, 2H, *J* = 8.8 Hz), 3.80 (s, 3H), 3.03–3.40 (m, 3H), 2.67–3.03 (m, 2H); ^13^C-NMR (63 MHz, CDCl_3_) δ 196.8 157.8 139.3 135.8 133.9 133.2 128.0 127.7 127.1 125.9 114.0 55.8 45.3 41.3 37.8; LC-MS (ESI+) *m/z* 273 [M+Na].

#### 3.3.2. 3-(4-Hydroxyphenyl)-l-tetralone (**12**)

Tetralone **9** (50 mg, 0.20 mmol) was added to HBr/HOAc (5 mL: 1/3 in volume) and refluxed for 6 h. The mixture was extracted by dichloromethane (30 mL) and washed with water (20 mL). The organic layer was dried over MgSO_4_, filtered, and evaporated under reduced pressure. The reaction mixture purified by flash column chromatography (silica gel, ethyl acetate/*n*-hexane = 1:2, v/v) to give pure product **12** (38 mg, 81%). Dark brown solid. mp 107–109 °C; IR (neat, KBr) ν_max_ 3350, 1605, 1505, 1250, 1025, 735 cm^−1^; ^1^H-NMR (CDCl_3_, 250 MHz) δ 7.62 (d, 1H, *J* = 7.4 Hz, aromatic-H), 7.28–7.19 (m, 4H, aromatic-H), 7.15 (d, 1H, *J* = 7.4 Hz, aromatic-H), 6.89 (d, 2H, *J* = 8.6 Hz, aromatic-H), 4.98 (dd, 1H, *J* = 10.3, 5.8 Hz, CH), 3.81 (s, 3H, CH_3_), 3.10–2.91 (m, 3H, CH_2_, OH), 2.50–2.43 (m, 1H, CH), 1.96–1.82 (m, 2H, CH_2_). ^13^C-NMR (Acetone D_6_, 63 MHz) δ 196.8, 156.1, 143.9, 134.9, 133.5, 132.2, 129.0, 127.7, 126.6, 126.4, 115.3, 45.9, 40.3, 37.6; LC-MS (ESI+) *m/z* 238 [M^+^] HRMS: *m/z* = 238.0985, calcd. 238.0994 for C_16_H_14_O_2_: [M+H]^+^). 

#### 3.3.3. 3-[1,2,3,4-Tetrahydro-3-[4-(4-trifluoromethylbenzyloxy)phenyl]-1-naphthalen-1-one (**13**)

Sodium hydride (14 mg, 0.60 mmol) was added into the solution of tetralone phenol **12** (100 mg, 0.40 mmol) in THF (5 mL) and the mixture was stirred for 30 min. at 0 °C. Then a freshly prepared 3-(trifluoromethyl)benzyl bromide (96 mg, 0.40 mmol) was added and the solution was stirred 1 h at 0 °C. The reaction mixture was washed with water (10 mL). The organic layer was separated, and washed with saturated aqueous NH_4_Cl solution (10 mL), and dried over anhydrous MgSO_4_, filtered, and concentrated under reduced pressure. The residue was purified by flash column chromatography (silica gel, ethyl acetate/*n*-hexanes = 1:4, v/v) to give the ketone **13** (116 mg, 70%). Yellow solid. mp 123–125 °C; IR (neat, KBr) ν_max_ 3079, 3025, 2931, 2889, 1671, 1597, 1512, 1454, 1327, 1252, 1165, 1119, 1067, 828, 761 cm^−1^. ^1^H-NMR (CDCl_3_, 400 MHz) δ 8.08 (dd, 1H, *J* = 8.4, 8.4 Hz, aromatic-H), 7.65 (d, 1H, *J* = 7.5 Hz, aromatic-H), 7.56 (d, 1H, *J* = 8.0 Hz, aromatic-H), 7.52–7.48 (m, 1H, aromatic-H), 7.35 (t, 1H, *J* = 8.5 Hz, aromatic-H), 7.30–7.23 (m, 2H, aromatic-H), 7.23 (d, 1H, *J* = 7.5 Hz, aromatic-H), 7.14 (d, 1H, *J* = 8.6 Hz, aromatic-H), 6.96 (d, 1H, *J *= 8.0 Hz, aromatic-H), 6.85 (d, 1H, *J* = 8.0 Hz, aromatic-H), 5.13 (s, 2H, benzyl-H), 3.47-3.34 (m, 1H, CH), 3.15 (t, 2H, *J* = 7.8 Hz, CH_2_), 2.98–2.90 (m, 1H, CH_2_), 2.84–2.74 (m, 1H, CH_2_). ^13^C-NMR (CDCl_3_, 100 MHz) δ 199.3, 158.6, 156.0, 144.5, 142.2, 137.4, 136.5, 134.8 (2C), 133.2, 129.9, 128.8 (2C), 128.4, 128.2, 128.0, 127.9, 126.6, 116.6, 116.1, 69.6, 46.7, 40.8, 38.4. LC-MS (ESI+) *m/z* 396 [M^+^] HRMS: *m/z* = 396.1342, calcd. 396.1337 for C_24_H_19_F_3_O_2_: [M+H]^+^).

#### 3.3.4. 3-[1,2,3,4-Tetrahydro-3-[4-(4-trifluoromethylbenzyloxy)phenyl]-1-naphthalen-1-ol (**5**)

To a stirred solution of ketone **13** (0.8 g, 2.0 mmol) in EtOH (6 mL) was added portionwise sodium borohydride (91 mg, 2.4 mmol) and then the mixture was stirred at room temperature for 2 h. The reaction mixture was diluted with water (5 mL) and acidified with 1 N HCl aqueous solution. The resulting mixture was extracted with dichloromethane (10 mL × 3). The combined organic layer was washed with saturated aqueous NH_4_Cl solution (15 mL) and organic phase was separated, dried over anhydrous MgSO_4_, filtered, and concentrated under reduced pressure. The residue was purified by flash column chromatography (silica gel, ethyl acetate/*n*-hexanes = 1:3, v/v) to afford alcohol **5** (0.56 g, 70%) as a white solid. R*_f_* = 0.3 (30% ethyl acetate/hexanes). mp 111.6 °C. IR (neat, NaCl) ν_max_ 3388 (OH), 2922 (C-H), 1611 (C=C), 1584, 1512, 1454, 1244, 1066 (C-O), 824 cm^−1^. ^1^H-NMR (CDCl_3_, 500 MHz) δ 7.63 (dd, 3H, *J* = 8.5, 8.5 Hz, aromatic-H), 7.56 (d, 2H, *J* = 8.0 Hz, aromatic-H), 7.29–7.20 (m, 4H, aromatic-H), 7.10 (d, 1H, *J* = 7.5 Hz, aromatic-H), 6.95 (d, 2H, *J* = 8.0 Hz, aromatic-H), 5.13 (s, 2H, benzyl-H), 5.02–4.96 (m, 1H, CH), 3.09–2.88 (m, 2H, CH_2_), 2.51–2.45 (m, 1H, CH), 1.92 (q, 1H, *J* = 12.5 Hz, CH_2_), 1.78 (d, 1H, *J* = 8.0 Hz, CH_2_). ^13^C-NMR (CDCl_3_, 125 MHz) δ 157.2, 141.4, 139.3, 138.4, 136.4, 128.7, 127.9 (2C), 127.6, 127.5 (2C), 126.9, 126.7, 125.7 (2C), 125.6 (2C), 115.1 (2C), 70.3, 69.4, 41.0, 38.7, 38.6. HRMS: *m/z* = 399.1585 (calcd. 399.1572 for C_24_H_22_F_3_O_2_: [M+H]^+^).

### 3.4. Synthesis of Flocoumafen (**1**)

A catalytic amount of *p*-toluenesulfonic acid (5 mg) and 4-hydroxycoumarin (0.1 g, 0.6 mmol) were added to a stirred solution of secondary alcohol (**5**, 0.2 g, 0.5 mmol) in dichloromethane (4 mL). The mixture was refluxed for 6 h. The reaction mixture was cooled to room temperature and was washed with water (5 mL). The organic layer was separated, and the aqueous layer was extracted with dichloromethane (10 mL × 3). The combined organic layers were washed with saturated aqueous NH_4_Cl solution (15 mL), and the organic phase was separated, dried over anhydrous MgSO_4_, filtered, and concentrated under reduced pressure. The residue was purified by flash column chromatography (silica gel, ethyl acetate/*n*-hexanes = 1:4, v/v) to give flocoumafen (**1**, 0.18 g, 64%) as a white solid. R*_f_* = 0.2 (25% ethyl acetate/hexanes). mp 137.5 °C. IR (neat, NaCl) ν_max_ 3396 (OH), 1668 (C=O), 1610 (C=C), 1570, 1510, 1452, 1419, 1326, 1240, 1066 (C-O), 825 cm^−1^. ^1^H-NMR (CDCl_3_, 500.14 MHz) δ 7.72 (d, 1/2H, *J* = 8.0 Hz, aromatic-H), 7.68–7.61 (m, 5/2H, aromatic-H), 7.57–7.50 (m, 3H, aromatic-H), 7.38–7.29 (m, 4H, aromatic-H), 7.28–7.23 (m, 2H, aromatic-H), 7.19 (dd, 2H, *J* = 8.5, 8.5 Hz, aromatic-H), 6.91 (dd, 2H, *J *= 8.5, 8.5 Hz, aromatic-H), 5.12 (s, 1H, benzyl-H), 5.10 (s, 1H, benzyl-H), 4.86 (q, 1/2H, *J *= 6.0 Hz, CH), 4.72 (t, 1/2H, *J *= 4.5 Hz, CH), 3.23 (d, 1/2H, *J *= 12.5 Hz, CH_2_), 3.13–2.98 (m, 5/2H, CH, CH_2_), 2.51–2.40 (m, 1/2H, CH_2_), 2.38–2.24 (m, 1/2H, CH_2_), 1.96–1.82 (m, 1/2H, CH_2_). ^13^C-NMR (CDCl_3_, 125.76 MHz) δ 163.5 (C=O), 160.8 (C), 157.2 (C), 157.1 (C), 152.7 (C), 152.6 (C), 141.3 (C), 138.1 (C), 137.9 (C), 137.7 (C), 134.3 (C), 132.1 (CH), 132.0 (CH), 130.8 (C), 130.7 (C), 130.6 (CH), 129.3 (CH), 128.7 (CH), 128.1 (CH), 128.0 (C), 127.9 (C), 127.8 (C), 127.5 (C), 127.4 (C), 125.7 (C), 125.6 (C), 124.1 (CH), 124.0 (CH), 123.2 (CH), 123.1 (CH), 116.6 (CH), 116.5 (CH), 116.3 (C), 115.1 (C), 115.0 (C), 109.4 (C), 108.8 (C), 69.3 (benzyl-C), 39.8(CH), 38.6 (CH_2_), 38.1 (CH_2_), 37.5 (CH), 37.0 (CH), 36.5 (CH_2_), 35.9 (CH_2_). HRMS calcd for C_33_H_26_F_3_O_4_ [M+H]^+^ 543.1783, found *m/z* 543.1772; *cis*-Flocoumafen: mp 180.1 °C. IR (neat, NaCl) ν_max_ 3405 (OH), 1664 (C=O), 1607 (C=C), 1078 (C-O), 830 cm^−1^; L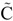-MS (ESI^+^) *m/z* 565.1 [M+Na]^+^, HRMS calcd for C_33_H_26_F_3_O_4_ [M+H]^+^ 543.1783, found *m/z* 543.1772; *trans*-Flocoumafen: mp 107.3 °C. IR (neat, NaCl) ν_max_ 3401 (OH), 1665 (C=O), 1612 (C=C), 1080 (C-O), 831 cm^-1^; LCMS (ESI^+^) *m/z * 565.3 [M+Na]^+^; HRMS calcd for C_33_H_26_F_3_O_4_ [M+H]^+^ 543.1783, found *m/z* 543.1772.

## 4. Conclusions

In conclusion, we have developed a concise and efficient seven step total synthesis of flocoumafen (**1**) starting from readily available 4-methoxybenzaldehyde via Knoevenagel condensation, intra-molecular ring cyclization, and coupling reactions. This method is useful for generating the core tetralone skeleton of 4-hydroxycoumarin derivatives and provides a generalized access to various warfarin type anticoagulants. In addition, *cis*- and *trans*-flocoumafen in pure form for the evaluation of anticoagulant activities were separated, and for the first time, their structures were characterized through 2D NMR analyses and computer simulation techniques.

## Supplementary Materials

Supplementary materials can be accessed at: http://www.mdpi.com/1420-3049/17/2/2091/s1.
