# Supplementary material for: Total Synthesis of Flocoumafen via Knoevenagel Condensation and Intramolecular Ring Cyclization: General Access to Natural Product
Source: Molecules. 2012 Feb 21;17(2):2091–102. doi: 10.3390/molecules17022091 (PMC6268178; doi:10.3390/molecules17022091)
Supplement: Supplementary file 1 [file molecules-17-02091-s001.pdf]

Electronic Supplementary Information (ESI)

Total Synthesis of Flocoumafen via Knoevenagel Condensation and Intramolcular Ring Cyclization, General Access to Natural Products

Jae-Chul Jung [1,†], Eunyoung Lim [2,†], Yongnam Lee [2], Dongguk Min [2], Jeremy Ricci [2], Oee-Sook Park [3,*] and Mankil Jung [2,*]

[1] Department of Neuroscience and Medical Research Institute, School of Medicine, Ewha Womans University, Seoul 158-710, Korea

[2] Department of Chemistry, Yonsei University, Seoul 120-749, Korea

[3] Department of Chemistry, Institute for Basic Sciences, College of Natural Sciences, Chungbuk National University, Cheongju 361-763, Chungbuk, Korea

† These authors contributed equally to this work.

* Authors to whom correspondence should be addressed; E-Mails: ospark@cbnu.ac.kr (O.-S.P.); mjung@yonsei.ac.kr (M.J.); Tel: +82-43-261-2283 (O.-S.P.); Fax: +82-43-267-2279 (O.-S.P.); Tel: +82-2-2123-2648 (M.J.); Fax: +82-2-364-7050 (M.J.).

Table of Contents

^{1}H-NMR and ^{13}C-NMR spectrum of compounds, **8**, **10**, **5**, and **1** S2

High-resolution 2D NMR analyses of *cis*- and *trans*- flocoumafen (FCF, **1**) S13

Computational details S23

Separation and purification of cis and trans forms of flocoumafen (**1**) via recrystallization S26

^{1}H-NMR and ^{13}C-NMR spectra of compounds, 8, 10, 5, and 1.

^{1}H-NMR spectrum of compound **8**

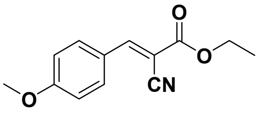

8

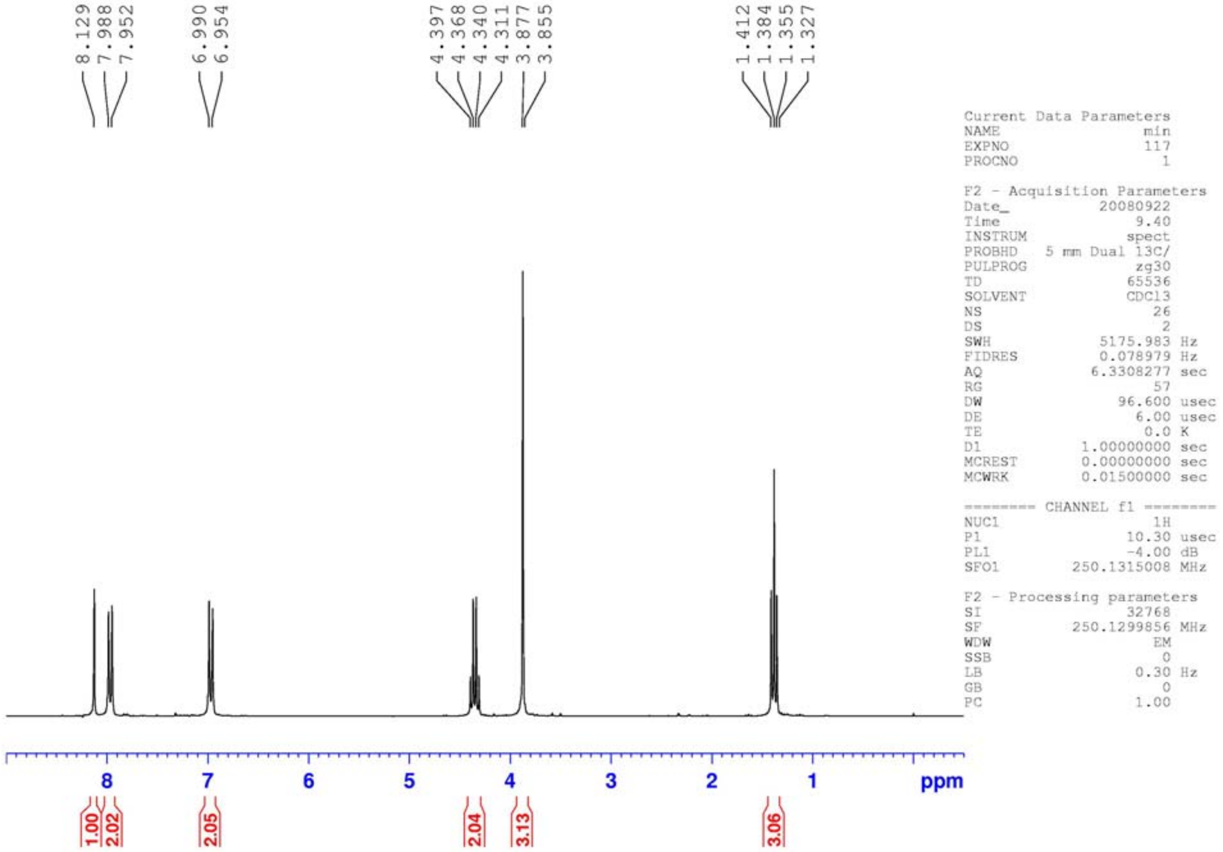

^{13}C-NMR spectrum of compound **8**

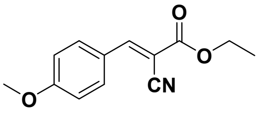

8

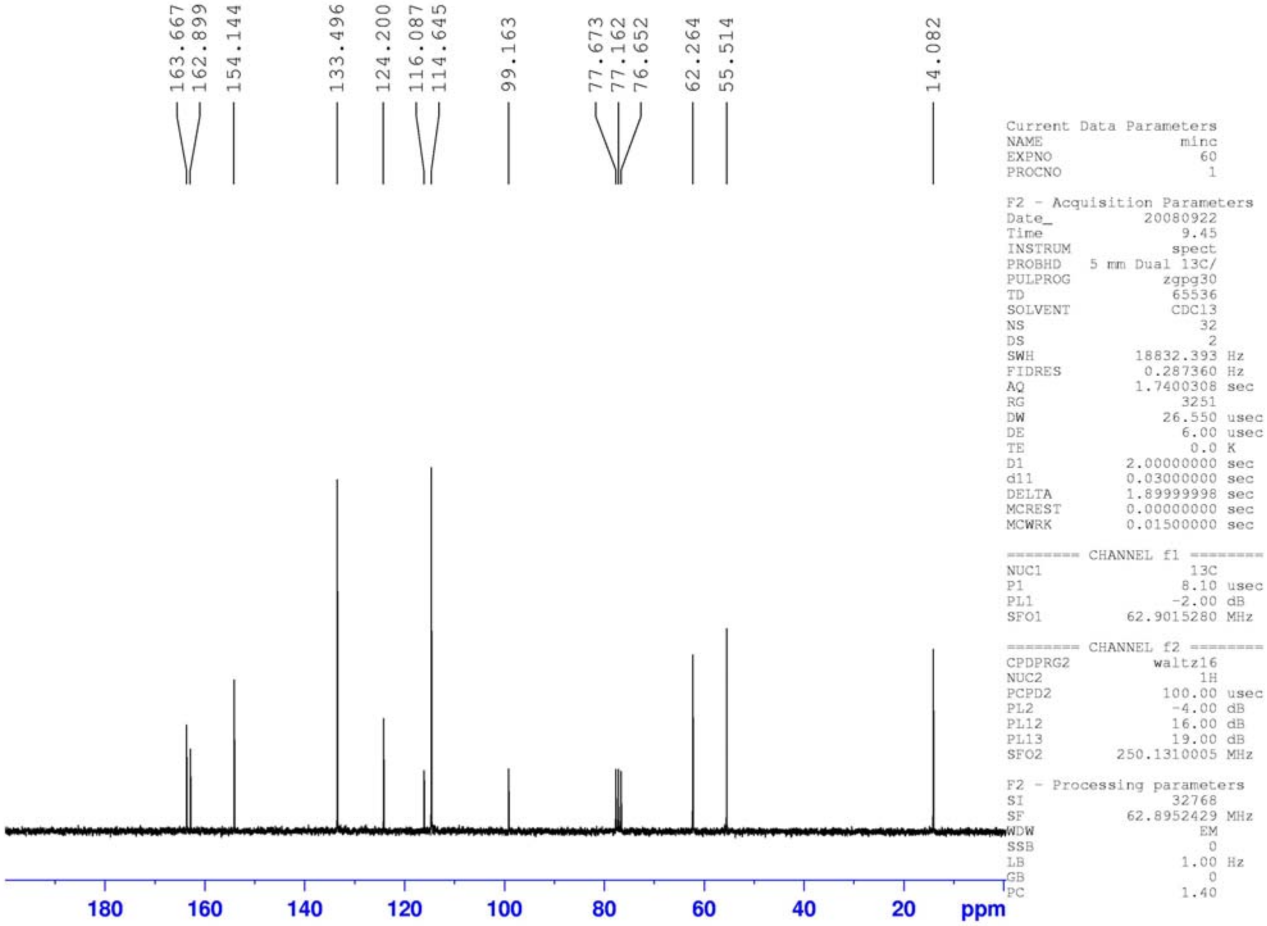

^{1}H-NMR spectrum of compound **10**

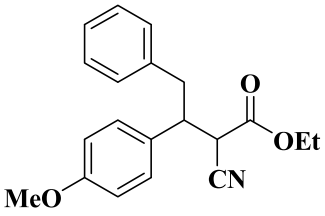

10

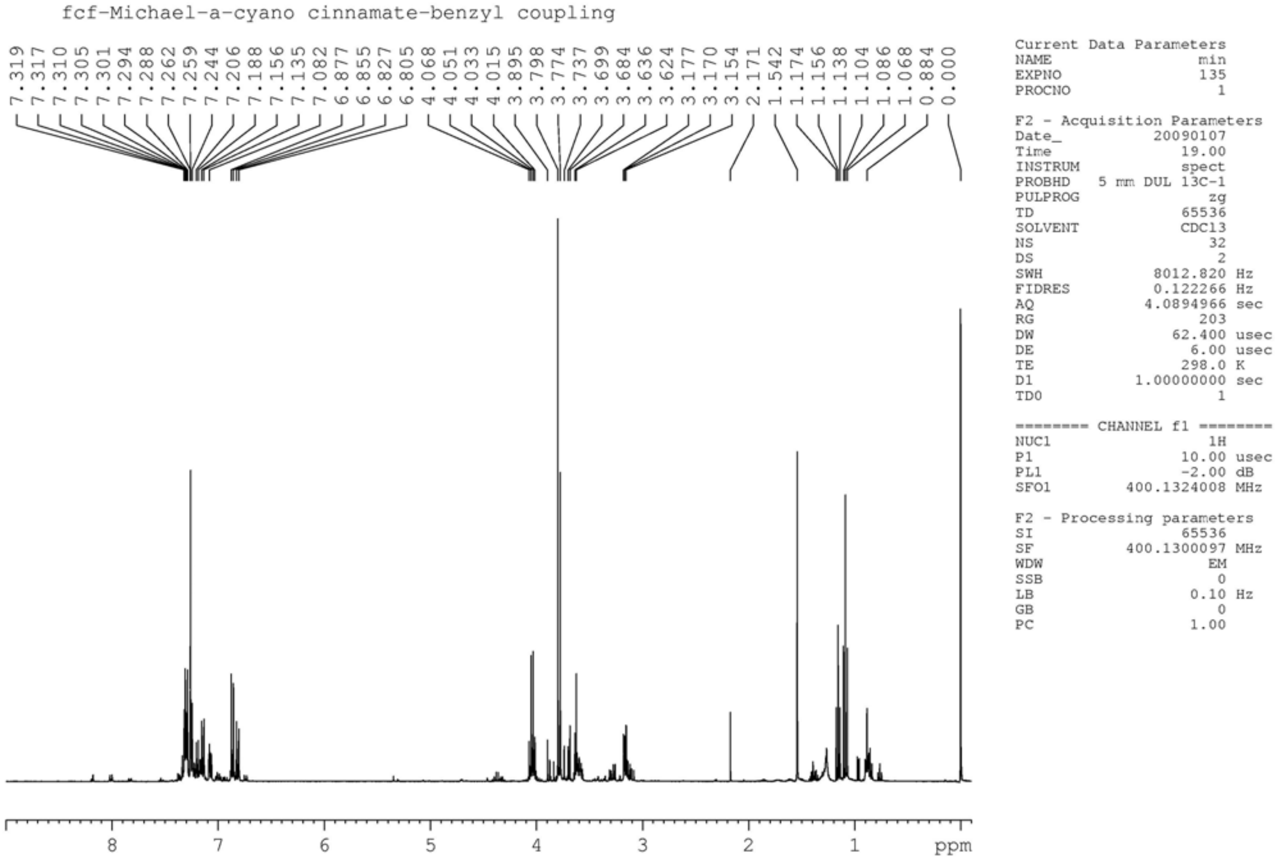

^{13}C-NMR spectrum of compound **10**

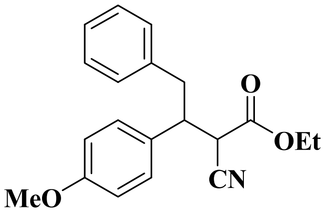

10

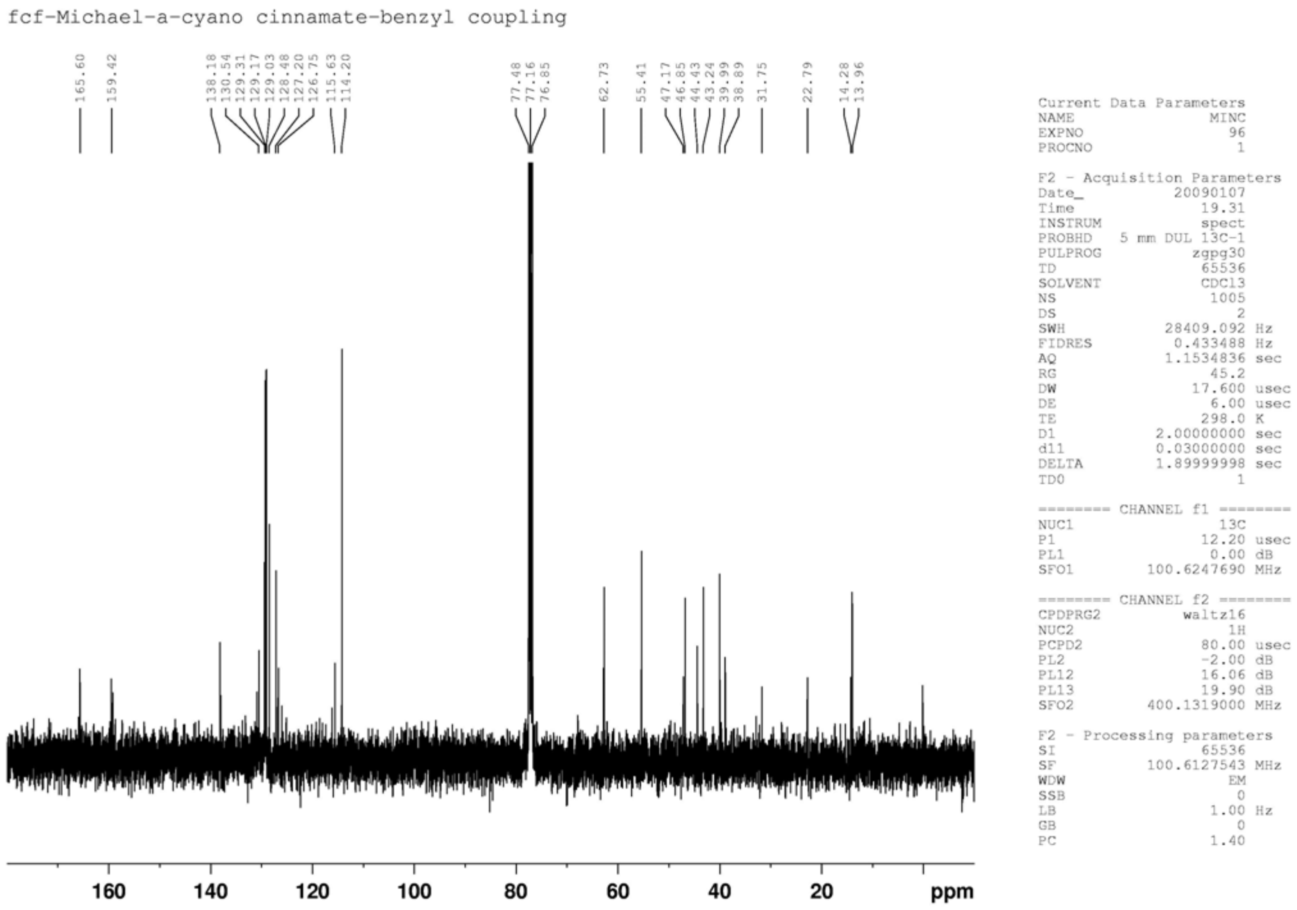

^{1}H-NMR spectrum of compound **5**.

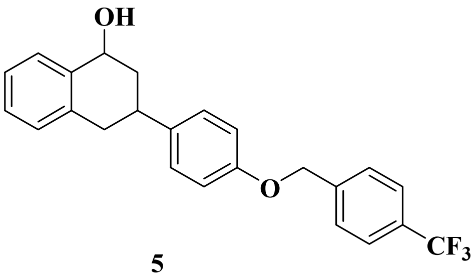

5

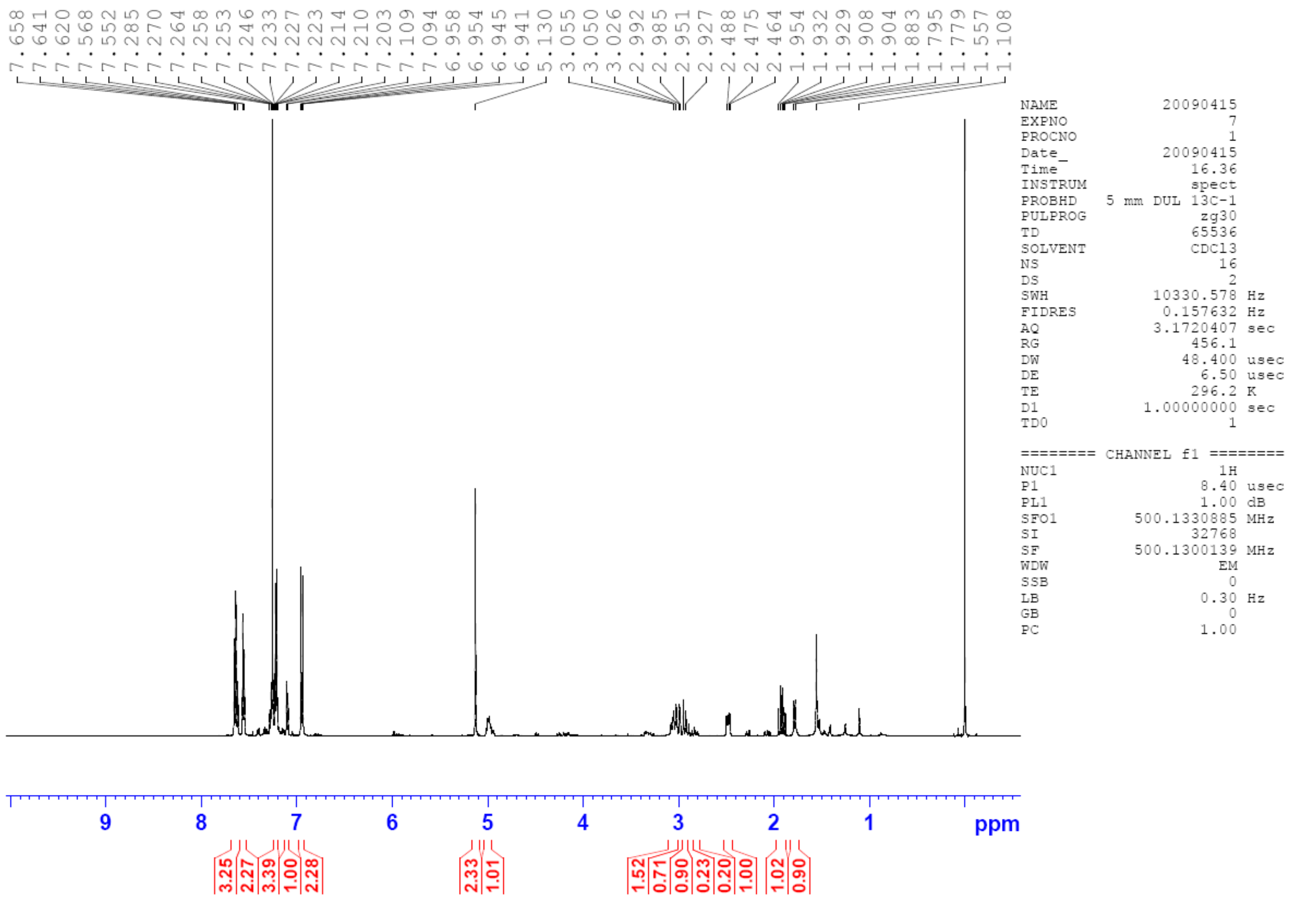

^{13}C-NMR spectrum of compound **5**.

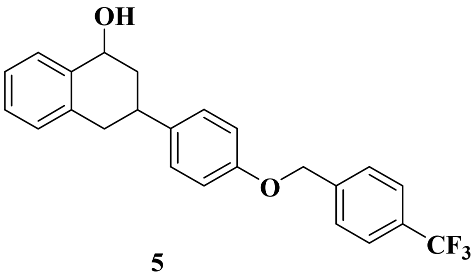

5

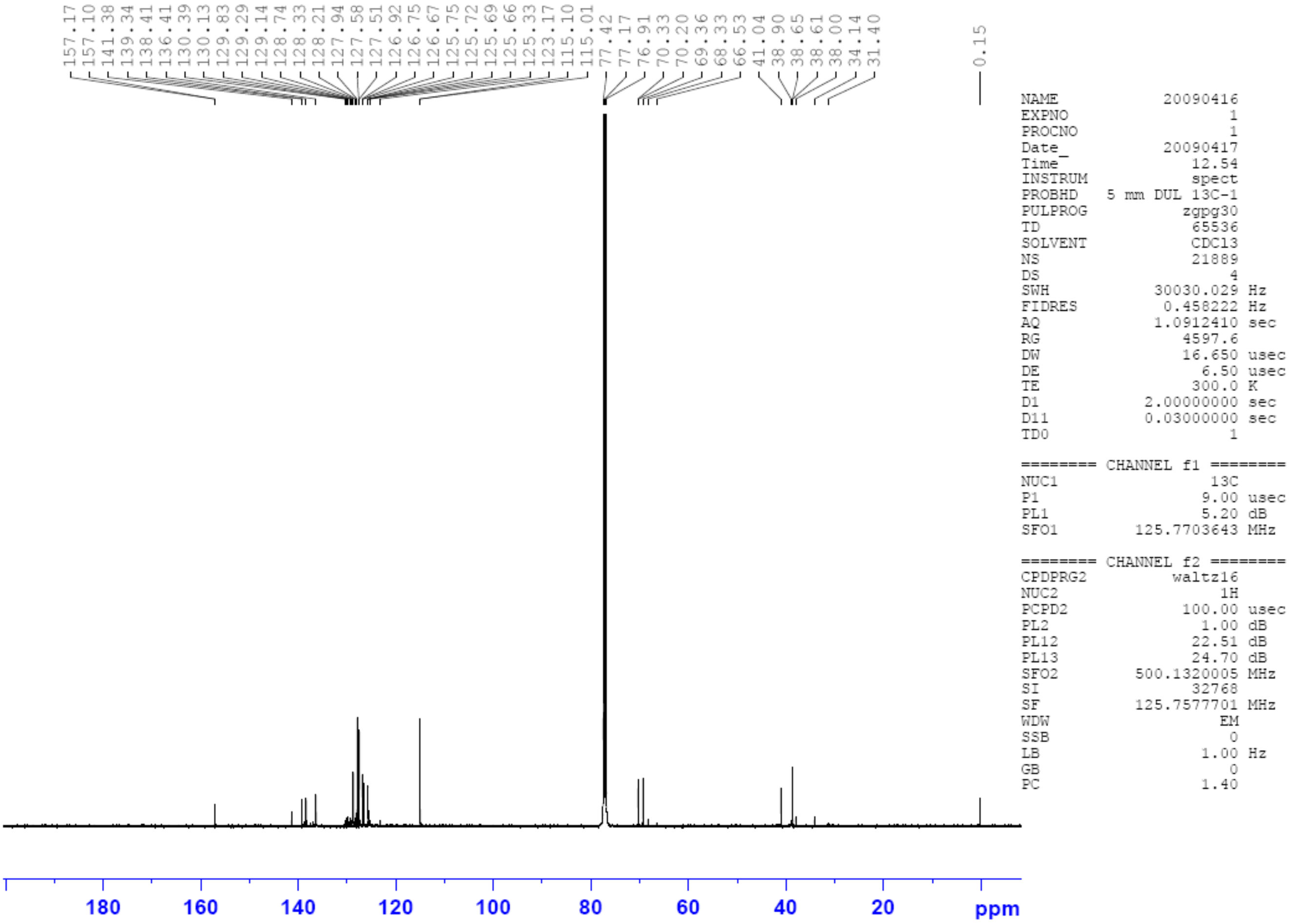

^{1}H-NMR spectrum of compound **1** (***cis*-FCF**)

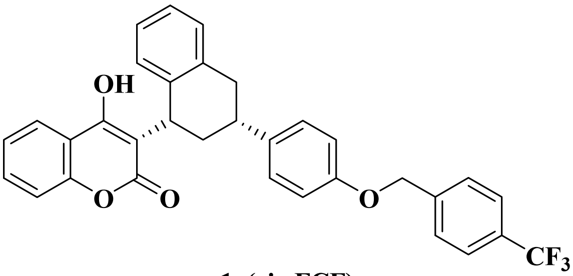

1, (cis-FCF)

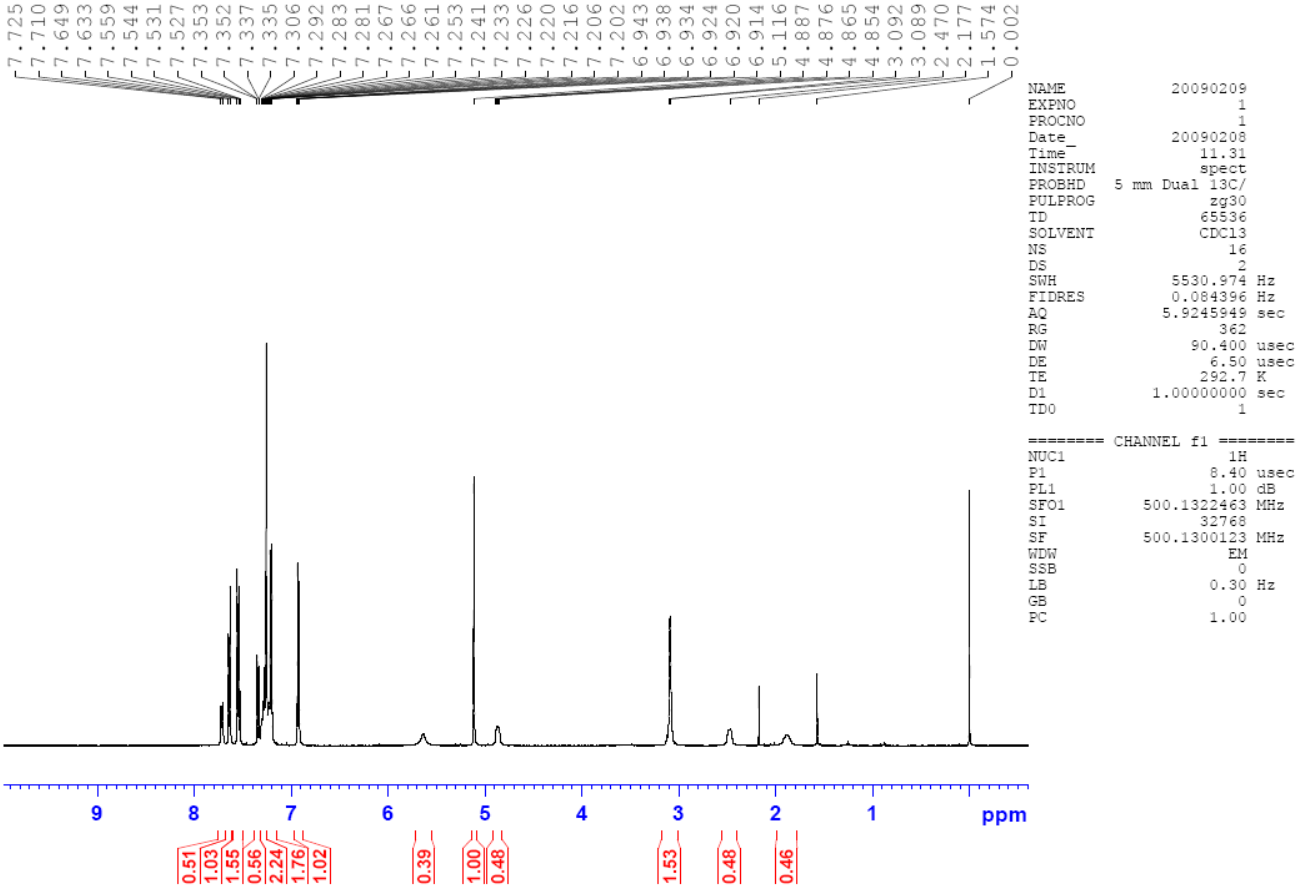

^{13}C-NMR spectrum of compound **1** (***cis*-FCF**)

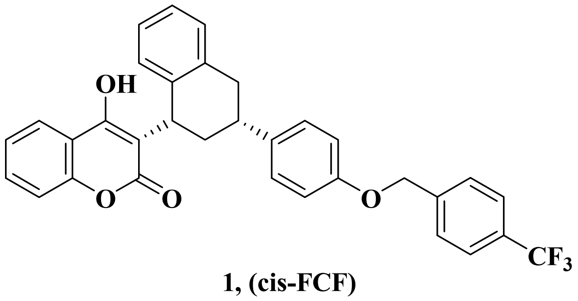

1, (cis-FCF)

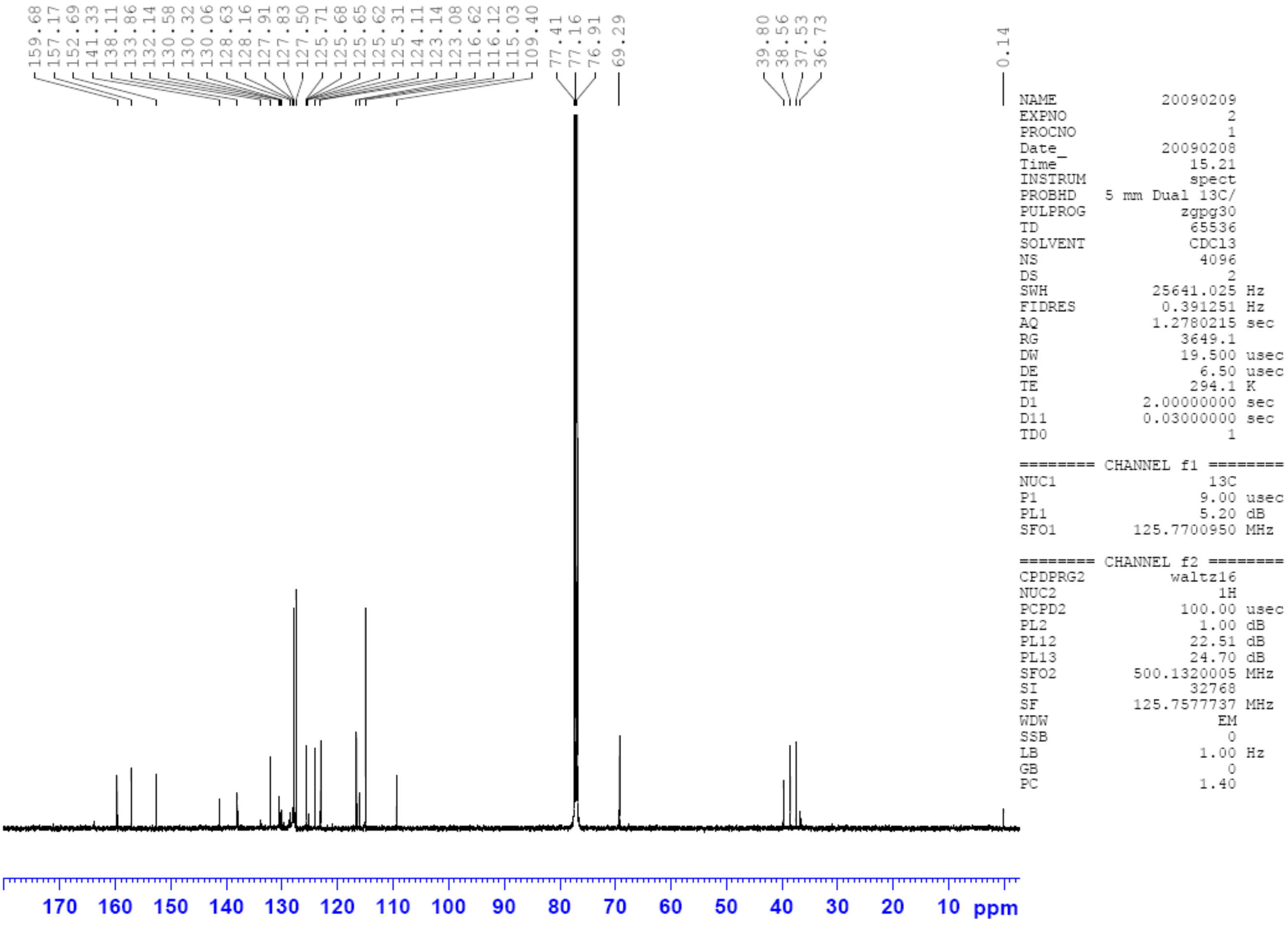

^{1}H-NMR spectrum of compound **1** (***trans*-FCF**)

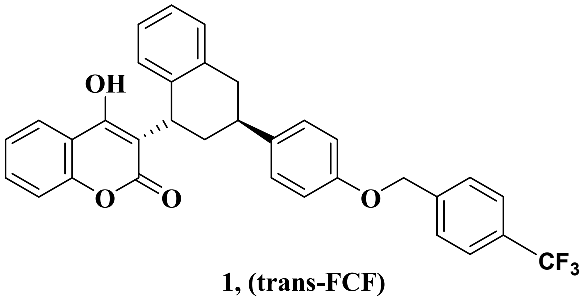

1, (trans-FCF)

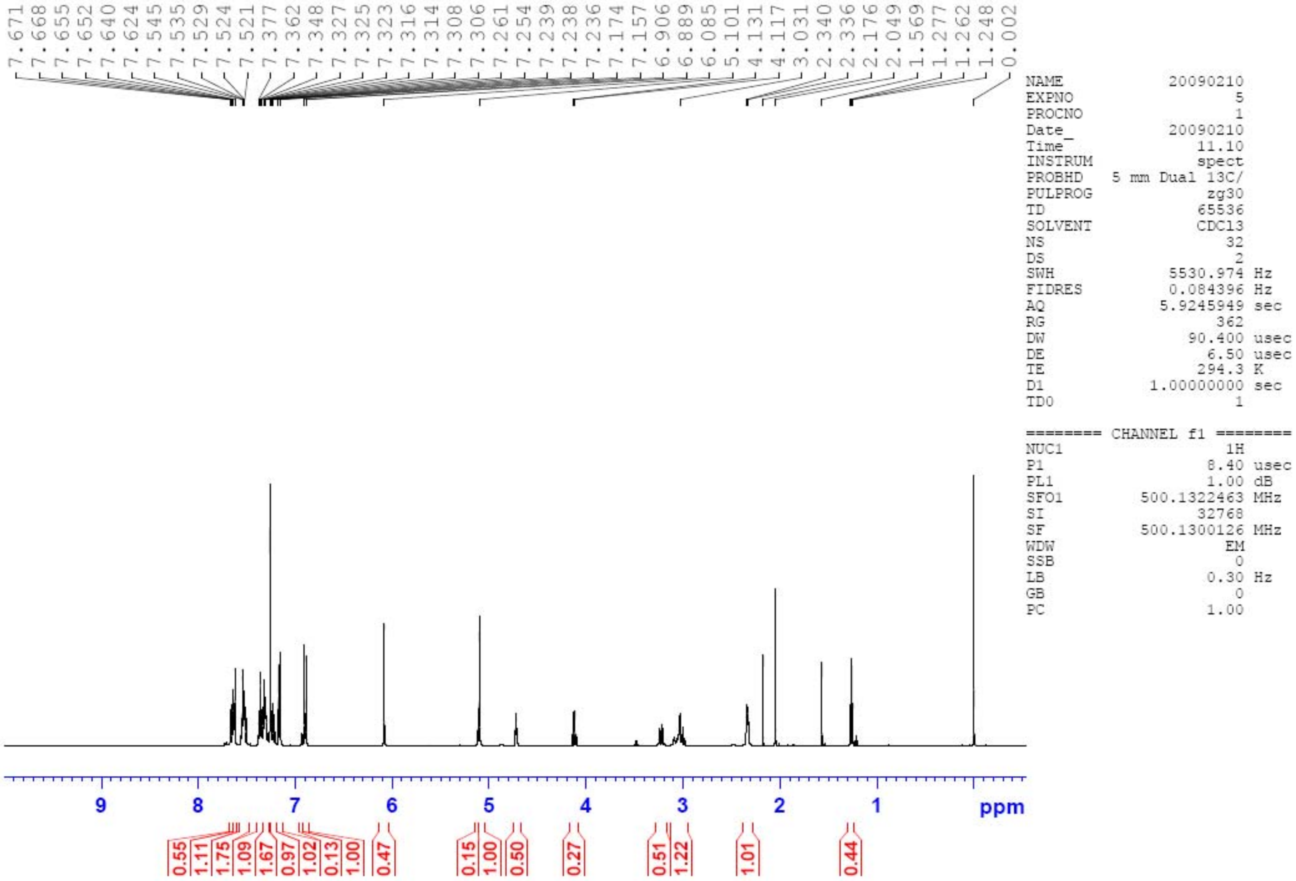

^{13}C-NMR spectrum of compound **1** (***trans*-FCF**)

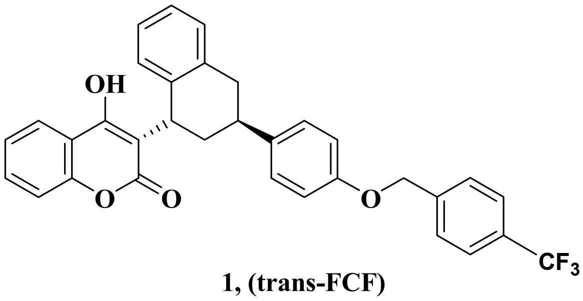

1, (trans-FCF)

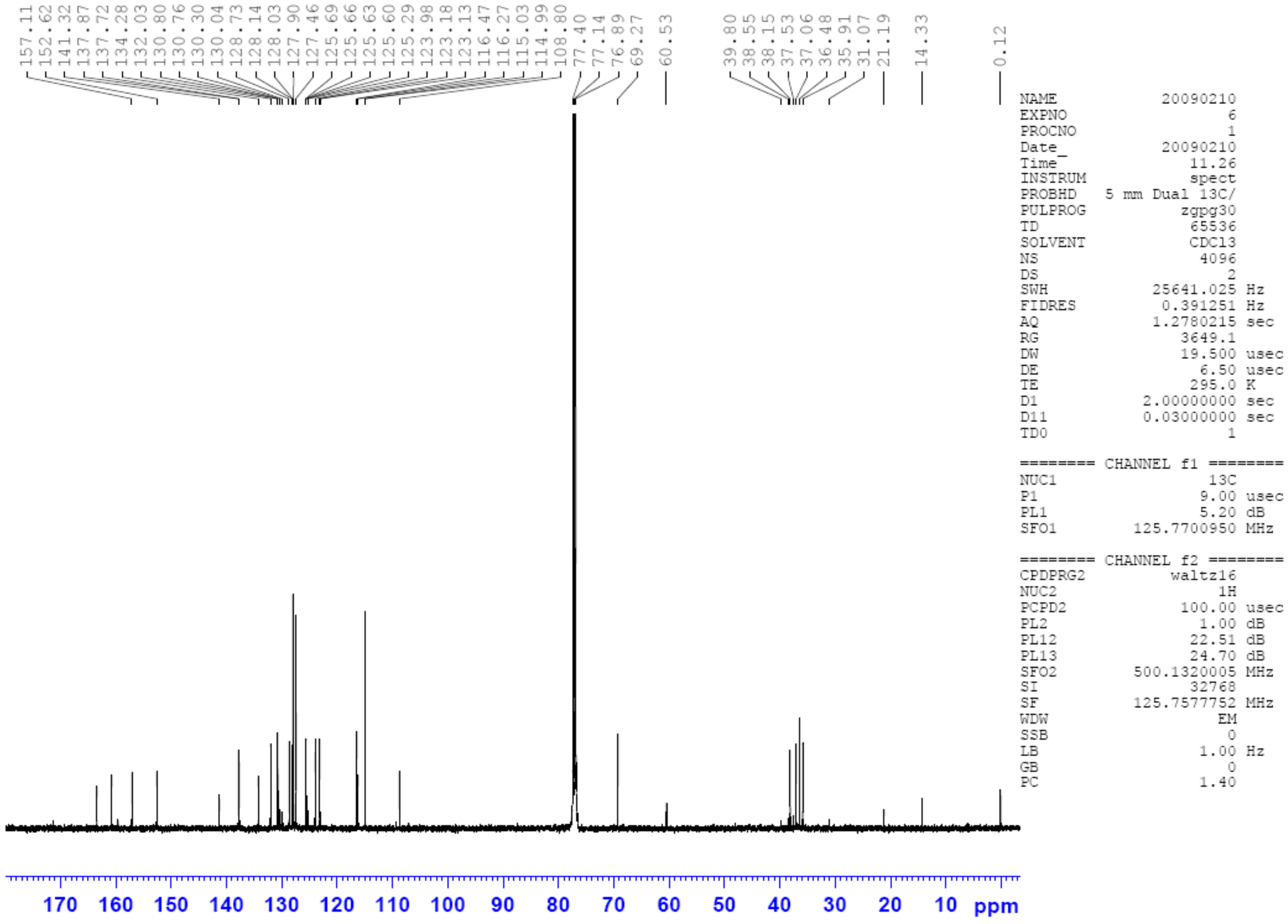

^{1}H-NMR spectrum of compound **1**, ***cis*-FCF *vs.* *trans*-FC**

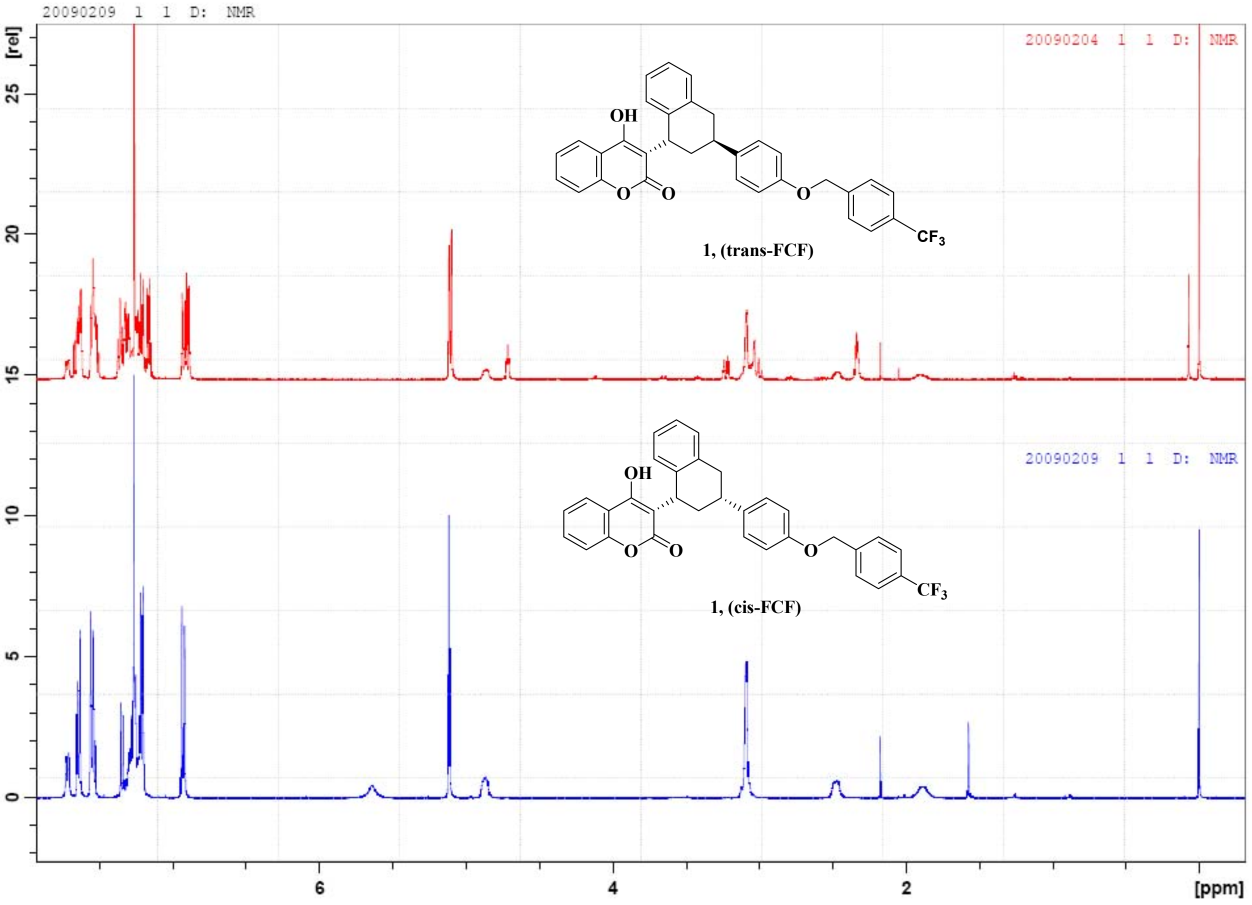

High–resolution 2D NMR analyses of *cis*- and *trans*- flocoumafen 1

COSY-NMR spectrum of compound **1** (***cis*-FCF**)

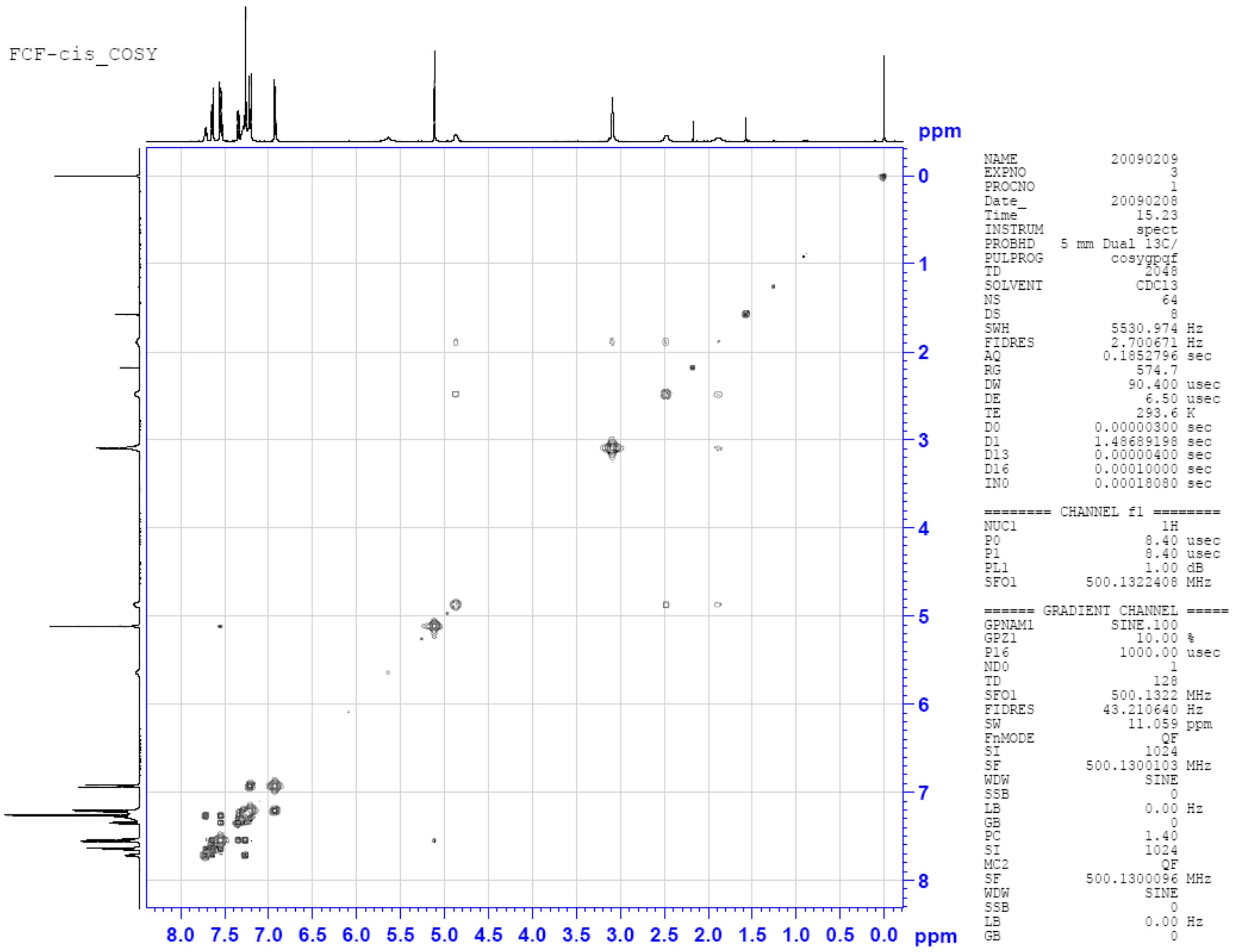

COSY-NMR spectrum of compound **1** (***trans*-FCF**)

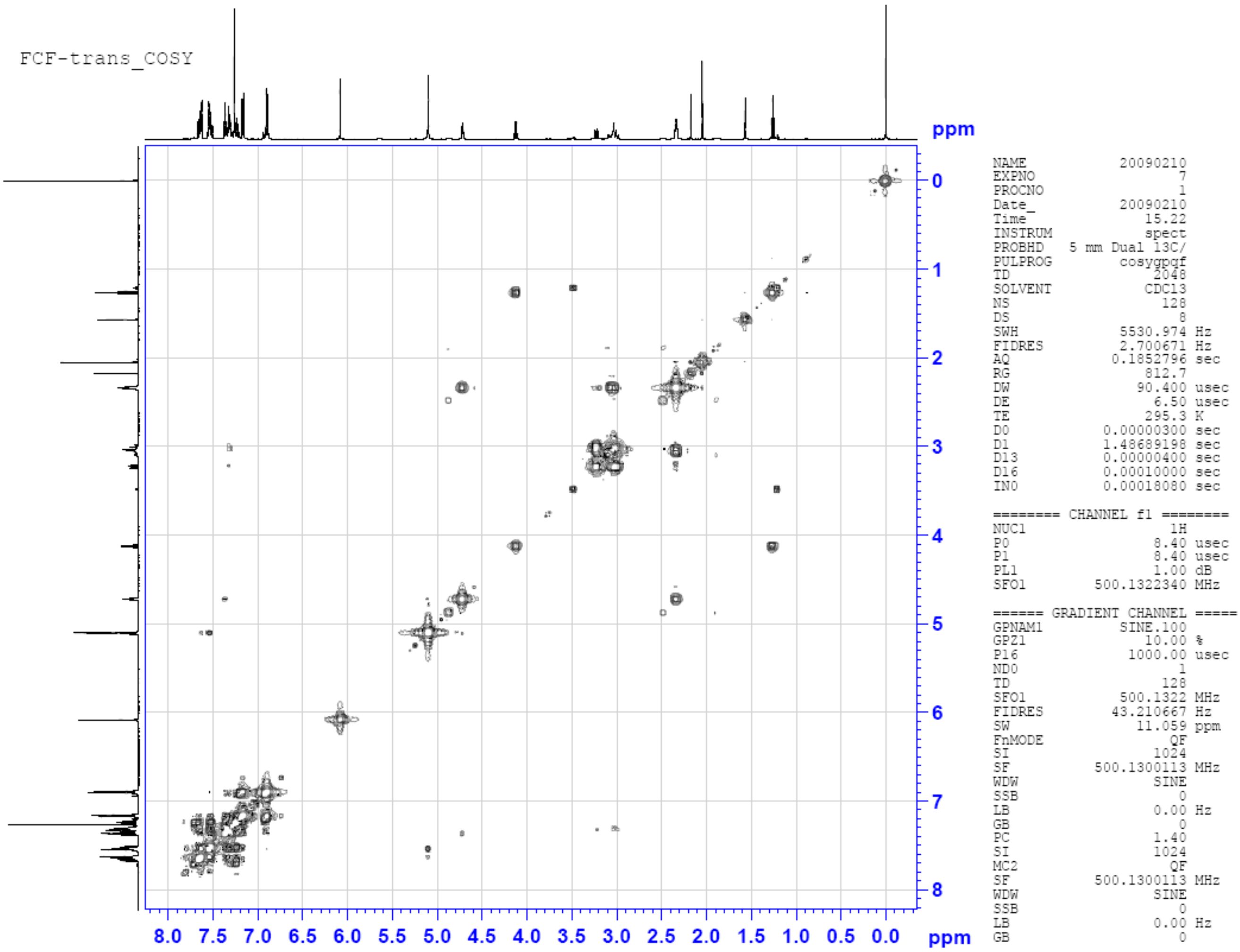

HMBC-NMR spectrum of compound **1** (***cis*-FCF**)

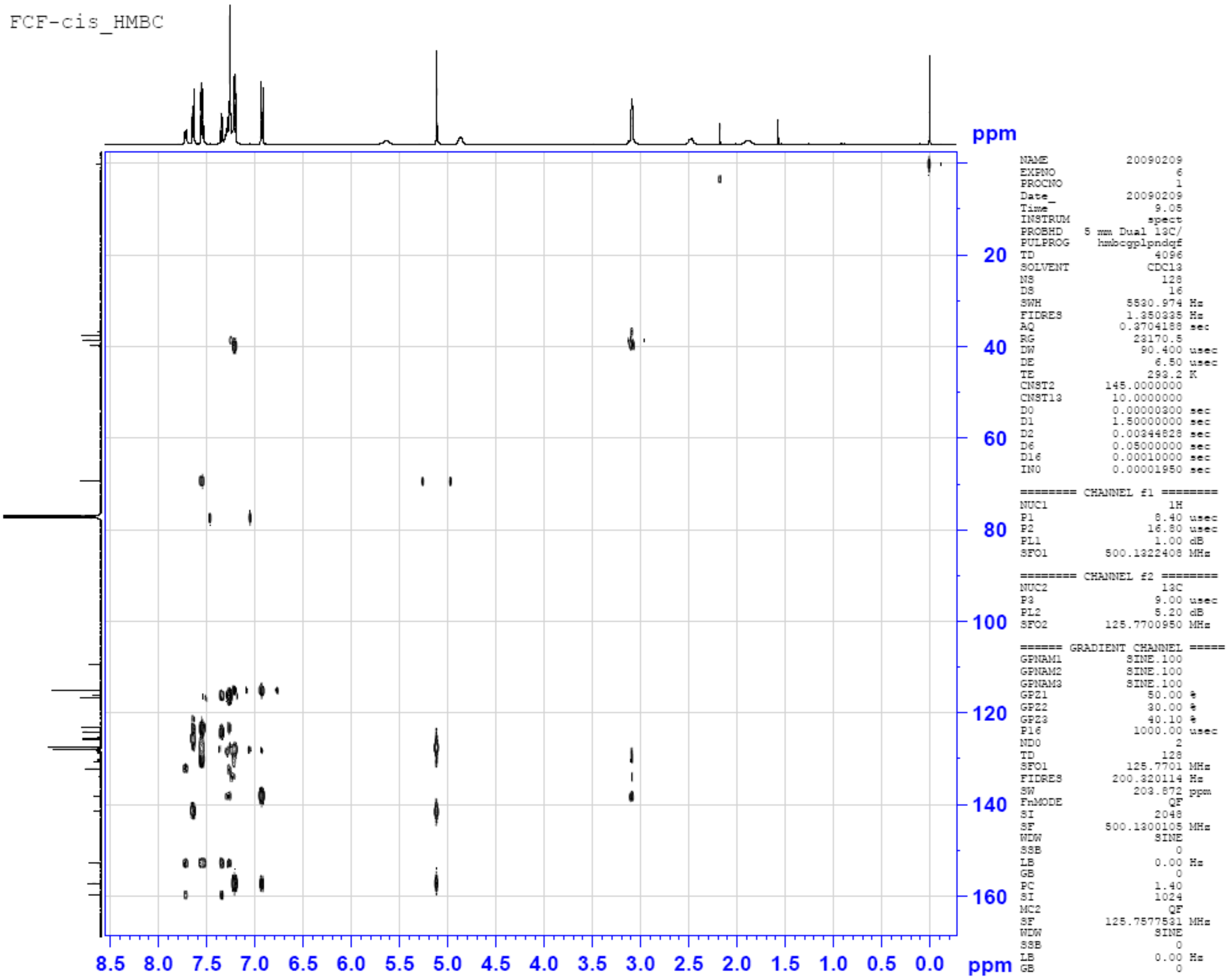

HMBC-NMR spectrum of compound **1** (***trans*-FCF**)

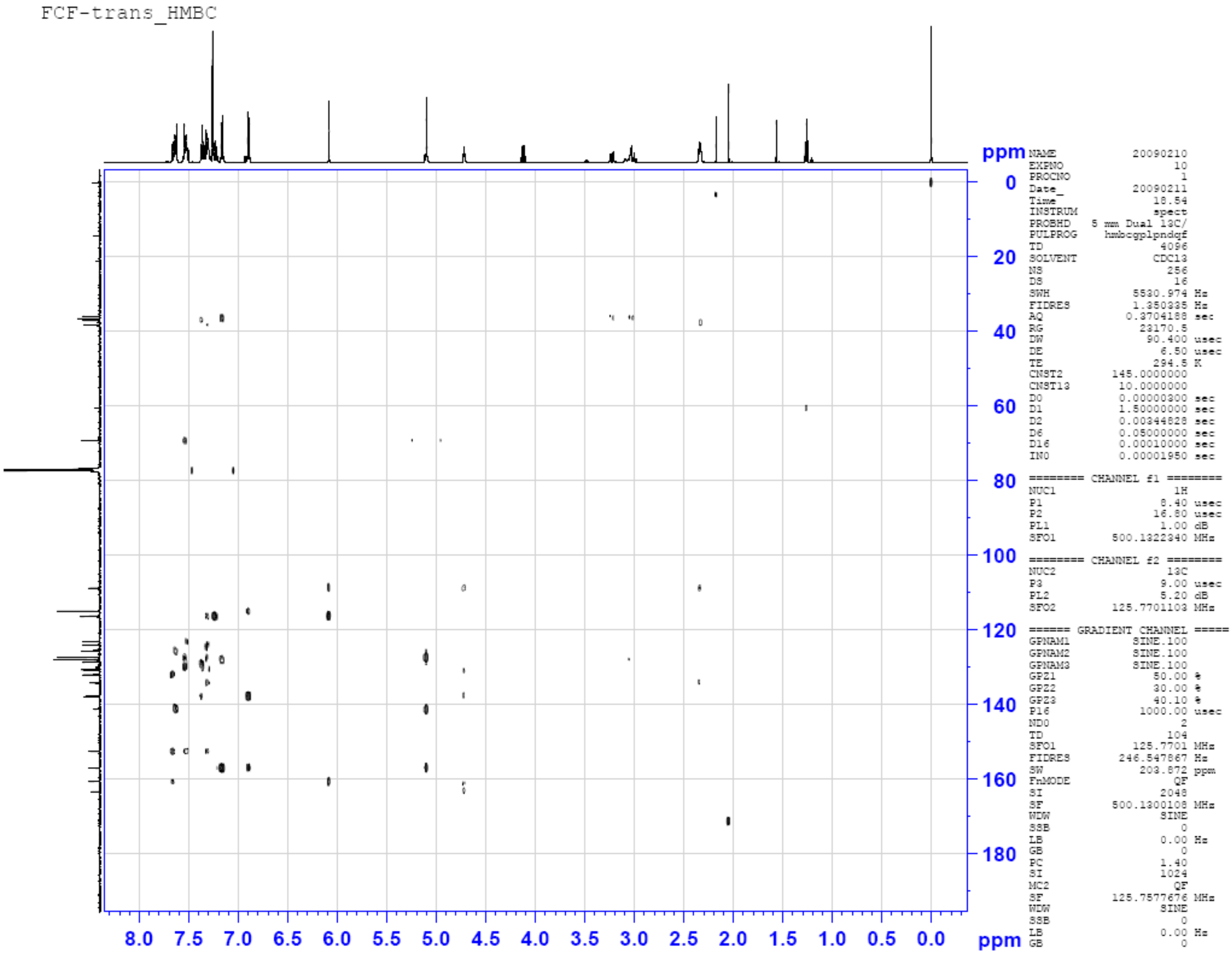

HMQC-NMR spectrum of compound **1** (***cis*-FCF**)

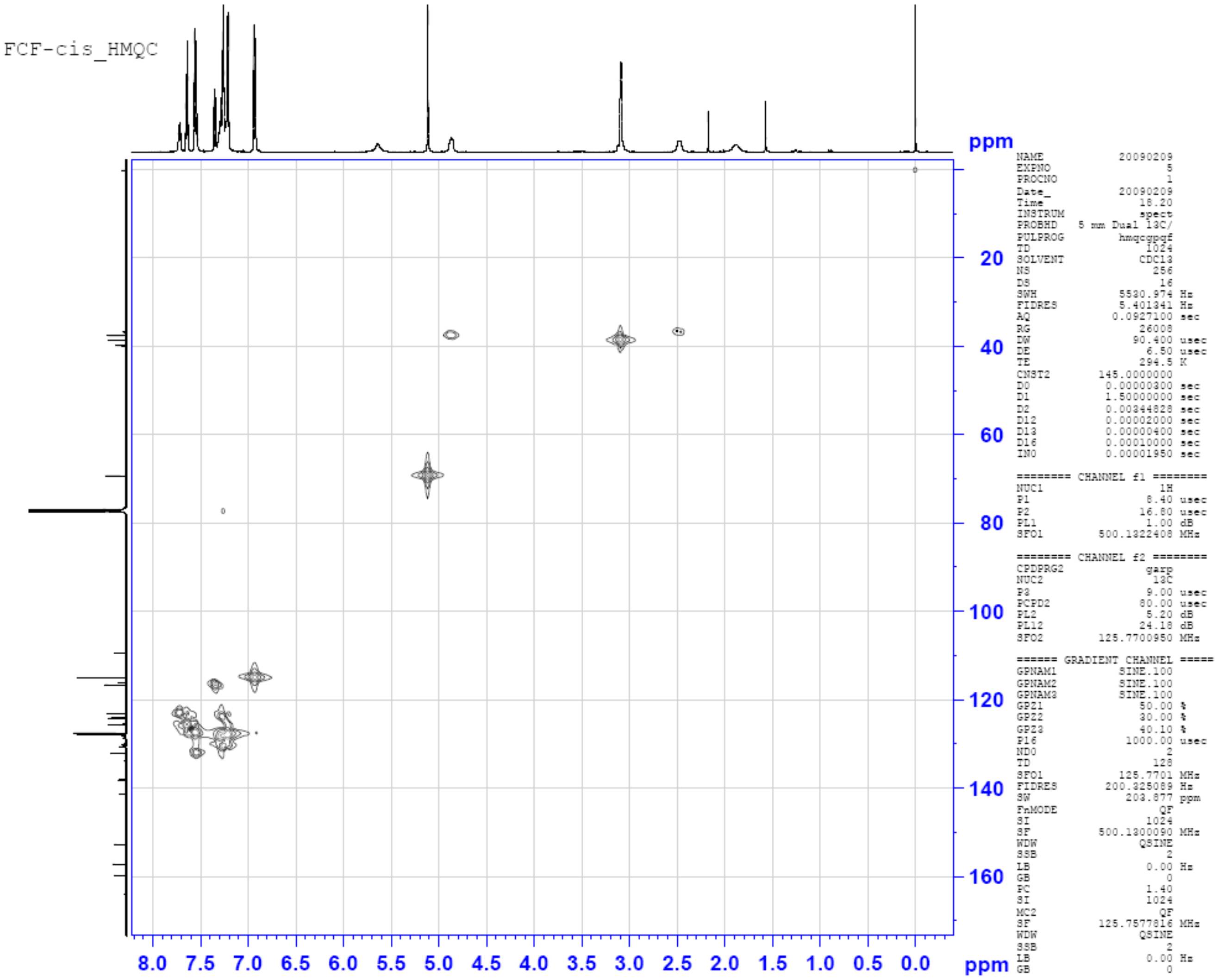

HMQC-NMR spectrum of compound **1** (***trans*-FCF**)

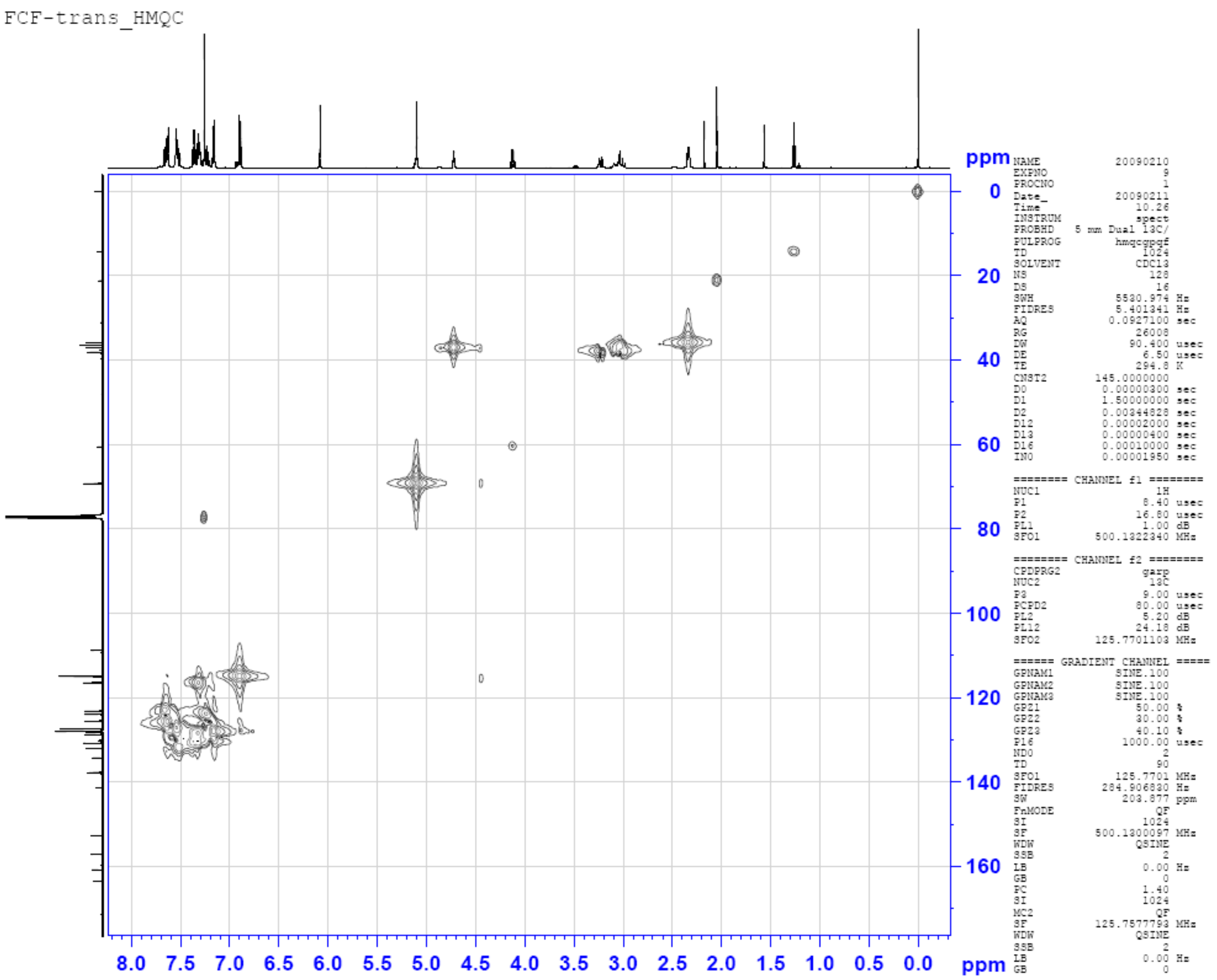

NOESY-NMR spectrum of compound **1** (***cis*-FCF**)

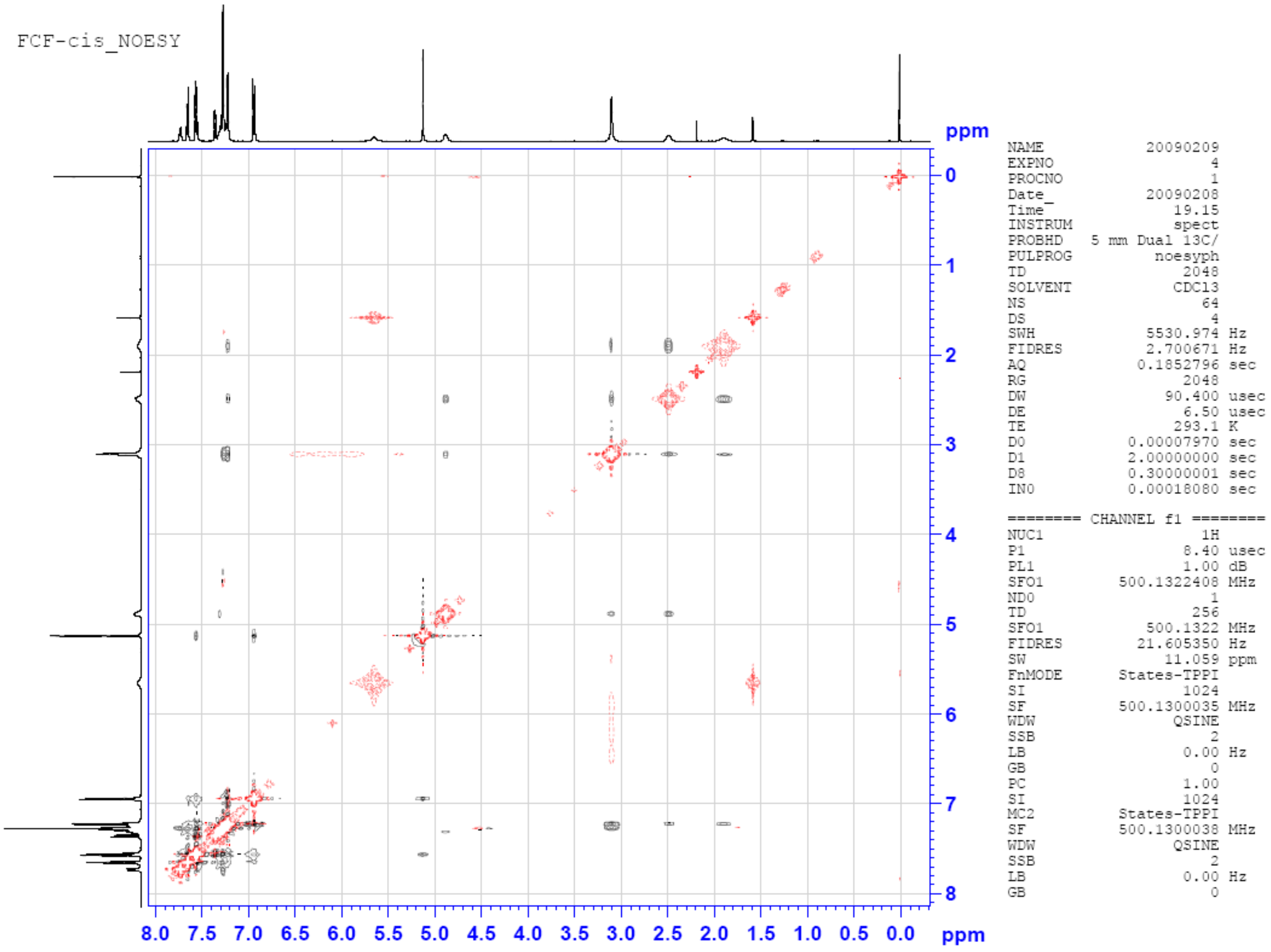

NOESY-NMR spectrum of compound **1** (***trans*-FCF**)

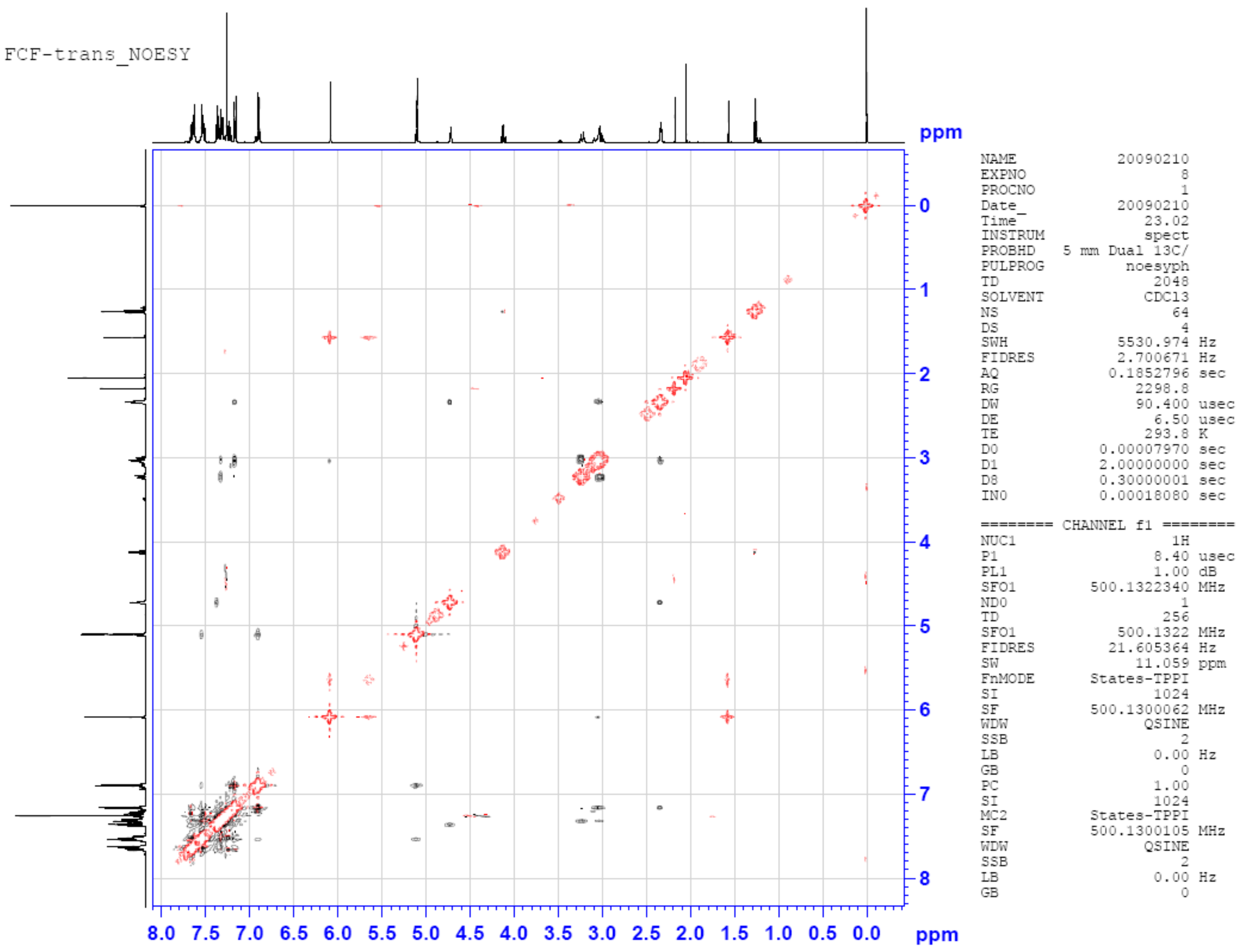

Table S1. ^{1}H-NMR (500 MHz) and ^{13}C-NMR (125 MHz) for *cis*-flocoumafen in $CDCl_3$.

Position	^{1}H (multi [a], *J* in Hz)	^{13}C [b]	HMBC [c]	NOESY [d]
2		163.8		
3		109.4		
4		159.7	5, 8, C	
4-OH	5.64			
5	7.72 (d, 7.5)	123.1	4, 7, 9, CH	H–6^{s}, H–7^{m}
6	7.32–7.23 (m)	124.1	8, 10, CH	H–5^{s}, H–7^{s}
7	7.53 (d, 7.5)	132.1	5, 9, CH	H–6^{s}, H–8^{s}, H–5^{w}
8	7.34 (d, 8.0)	116.6		
9		152.7	5, 7, 8, C	
10		116.1	6, 8, C	
1′	4.87 (dd, 5.5, 5.5)	37.5		H–2′^{m}, H–3′^{w}
2′	2.52–2.42 (m)<br>1.95–1.80 (m)	36.7	3′, 4′, CH_2	H–1′^{m}, H–3′^{m}, H–2″^{w}, H–6″^{w}
3′	3.13–3.02 (m)	39.8	2″, 6″, 4′, 10′, CH	H–2″^{s}, H–6″^{s}, H–2′^{m}, H–1′^{w}
4′	3.13–3.02 (m)	38.6	5′, 3′, CH_2	H–2′^{s}, H–1′^{w}, H–2″^{w}, H–6″^{w}
5′	7.32–7.23 (m)	130.1	4′, 7′, CH	H–6′^{s}, H–4′^{m}
6′	7.32–7.23 (m)	128.6		H–5′^{s}, H–7′^{s}
7′	7.32–7.23 (m)	128.2		H–6′^{s}, H–8′^{s}
8′	7.32–7.23 (m)	130.3		H–7′^{s}
9′		138.1	3″, 5″, 4′, C	
10′		138.0	3″, 5″, 3′, C	
1″		133.9	2″, 6″, C	
2″	7.21 (d, 9.0)	127.9		H–3″^{s}
3″	6.92 (d, 9.0)	115.0		H–2″^{s}
4″		157.2	2″, 6″, 3″, 5″,<br>benzyl, C	
5″	6.92 (d, 9.0)	115.0		H–6″^{s}
6″	7.21 (d, 9.0)	127.9		H–5″^{s}
OCH_2-Ph	5.12 (s)	69.3	1‴, 2‴, 6‴, 4″,<br>CH_2	H–3‴^{m}, H–5‴^{m}, H–2‴^{w}, H–6‴^{w}
OBn		141.3	3‴, 5‴, benzyl, C	
ortho	7.53 (d, 7.5)	127.8		OBn (m)^{m}
meta	7.62 (d, 8.0)	125.6,<br>125.7		OBn (o)^{m}
para		130.6		
CF_3		125.3	3‴, 5‴, C	

[a] Multi., multiplicity: s, singlet; d, doublet; t, triplet; q; quartet; dd, doublet of doublet; m, multiplet; [b] The chemical shifts were extracted from ^{3}C and HMQC experiments; [c] The correlations were assigned as quaternary, tertiary and secondary carbons from HMBC and DEPT (135) analysis; [d] NOESY intensities are marked as strong (s), medium (m), and weak (w).

Table S2. ^{1}H-NMR (500 MHz) and ^{13}C-NMR (125 MHz) for *trans*-flocoumafen in $CDCl_3$.

Position	^{1}H (multi [a], *J* in Hz)	^{13}C [b]	HMBC [c]	NOESY [d]
2		163.4		
3		108.8		
4		160.7	5, 8, C	
4-OH				
5	7.66 (dd, 1.5, 1.5)	123.9	7, CH	H–6^s, H–7^m
6	7.27–7.20 (m)	124.0	8, CH	H–5^s, H–7^s
7	7.57–7.52 (m)	132.0	5, 9, CH	H–6^s, H–8^s, H–5^w
8	7.33–7.29 (m)	116.5	10, CH	H–7^m H–6^w
9		152.6	5, 7, 8, C	
10		116.3	8, C	
1′	4.72 (t, 4.0)	37.5	3′, CH	H–2′^{m}, H–8′^{w}
2′	2.36–2.32 (m)	35.9	4′, CH_2	H–1′^{m}, H–3′^{m}, H–4′^{w}
3′	3.12–2.99 (m)	36.5	2″, 6″, 4′, CH	H–2″^{s}, H–6″^{s}, H–2′^{m}, H–1′^{w}
4′	3.23 (d, 12.0)	39.8	1″, 3′, 5′, 9′, CH_2	H–2′^{s}, H–5′^{m}, H–2″^{m}, H–6″^{m}, H–1′^{w},
5′	7.33–7.29 (m)	128.7	4′, 7′, CH	H–6′^{s}, H–4′^{m}
6′	7.39–7.34 (m)	128.1		H–5′^{s}, H–7′^{s}
7′	7.27–7.20 (m)	127.9	5′, 9′, CH	H–6′^{s}, H–8′^{s}
8′	7.39–7.34 (m)	130.7		H–7′^{s}, H–1′^{w}
9′		137.9	3″, 5″, 4′, C	
10′		137.7	3″, 5″, C	
1″		134.3	2″, 6″, C	
2″	7.16 (d, 8.5)	128.0		H–3″^{s}, H–2′^{w}
3″	6.90 (d, 8.5)	115.0		H–2″^{s}
4″		157.1	2″, 6″, benzyl, C	
5″	6.90 (d, 8.5)	115.0		H–6″^{s}
6″	7.16 (d, 8.5)	128.0		H–5″^{s}, H–2′^{w}
OCH_2-Ph	5.12 (s)	69.3	1‴, 2‴, 6‴, 4″, CH_2	H–3‴^{m}, H–5‴^{m}, H–2‴^{w}, H–6‴^{w}
OBn		141.3	3‴, 5‴, benzyl, C	
ortho	7.57–7.52 (m)	127.5		OBn (m)^m
meta	7.63 (d, 8.0)	125.6, 125.7		OBn (o)^m
para		130.8		
CF_3		125.3	3‴, 5‴, C	

[a] Multi., multiplicity: s, singlet; d, doublet; t, triplet; q; quartet; dd, doublet of doublet; m, multiplet; [b] The chemical shifts were extracted from ^{3}C and HMQC experiments; [c] The correlations were assigned as quaternary, tertiary and secondary carbons from HMBC and DEPT (135) analysis; [d] NOESY intensities are marked as strong (s), medium (m), and weak (w).

Computational details

The lower energy for the *cis*/*trans* conformers, flocoumafens were searched the semi-empirical AM1 method. The lower energy conformers were submitted to a geometry optimization and energy calculations by density functional theories (DFT) model calculation at the B3LYP 6-31G** level. Molecular modeling was performed by using the SPARTAN 06 for Windows software package.

***cis*-flocoumafen (FCF): −1873.06139 au**

	**Cartesian Coordinates (Angstroms)**		
**Atom**	**X**	**Y**	**Z**
1 H	−6.9353011	4.6009024	1.6090860
2 C	−6.8159670	4.0889098	0.6607277
3 C	−6.4708353	2.7086555	−1.7575650
4 C	−6.1400002	2.8668623	0.6350600
5 C	−7.3133211	4.6110046	−0.5273922
6 C	−7.1421891	3.9227208	−1.7379614
7 C	−5.9587692	2.1639172	−0.5663485
8 H	−7.8394784	5.5606222	−0.5141897
9 H	−7.5358153	4.3392031	−2.6595563
10 H	−6.3310851	2.1610073	−2.6824940
11 O	−5.6686589	2.3866706	1.8193991
12 C	−4.9670094	1.1855089	1.9130643
13 O	−4.5591607	0.8734530	3.0122925
14 C	−5.2479686	0.9031442	−0.5004778
15 O	−5.0951940	0.2791793	−1.6876135
16 H	−4.8031940	−0.6373040	−1.5367405
17 C	−4.7803879	0.4129877	0.6906021
18 C	−3.9398698	−0.8488205	0.8262367
19 C	−1.5968972	−1.8018820	0.4633332
20 C	−2.0978399	−2.6784899	−0.6992221
21 C	−2.4594764	−0.5293504	0.5196603
22 H	−1.7632066	−2.3583067	1.3963936
23 H	−2.3886955	−0.0006226	−0.4401683
24 H	−3.9944745	-1.1176096	1.8879940
25 H	−1.5749731	-3.6416902	−0.6986004
26 H	−2.0869583	0.1565978	1.2862677
27 C	−4.4668550	−2.0511552	0.0351582
28 C	−3.5959905	−2.9170790	−0.6591940
29 C	−5.8420371	−2.3487246	0.0581183
30 H	−6.5104856	−1.6884488	0.6036662
31 C	−6.3572460	−3.4599109	−0.6015164
32 H	−7.4230544	−3.6647342	−0.5679691
33 C	−5.4947844	−4.3077297	−1.2996920
34 H	−5.8821018	−5.1796007	−1.8185098
35 C	−4.1309450	−4.0339531	−1.3192066
36 H	−3.4554772	−4.7005593	−1.8501021

37 C	−0.1100074	−1.5083172	0.3668075
38 C	2.6616703	−0.9760435	0.2154146
39 C	0.4310871	−0.7672743	−0.6887878
40 C	0.7771957	−1.9796863	1.3463881
41 C	2.1408277	−1.7223042	1.2795286
42 C	1.7995218	−0.4993491	−0.7775648
43 H	-0.2183916	−0.3787799	−1.4690215
44 H	0.3901779	−2.5580354	2.1816072
45 H	2.8209744	−2.0878148	2.0420201
46 H	2.1724260	0.0742262	−1.6177760
47 O	4.0158080	−0.7748973	0.2369467
48 C	4.5979688	0.0314549	−0.7775454
49 H	4.0941689	1.0102238	−0.8091474
50 H	4.4667608	−0.4353351	−1.7642157
51 C	6.0669891	0.2209249	−0.4795703
52 C	8.7784530	0.6852527	0.0442769
53 C	6.5474865	0.1986842	0.8339371
54 C	6.9605271	0.4689947	−1.5275092
55 C	8.3084479	0.7056279	−1.2707561
56 C	7.8962497	0.4267406	1.0958138
57 H	5.8611289	−0.0137626	1.6454737
58 H	6.6028338	0.4726237	−2.5539618
59 H	8.9977321	0.8901503	−2.0874508
60 H	8.2662163	0.3966451	2.1150561
61 C	10.2210390	0.9992336	0.3295044
62 F	10.6560400	0.3972082	1.4590420
63 F	11.0323376	0.6066696	−0.6786419
64 F	10.4189761	2.3291180	0.4930310
65 H	−1.8271457	−2.1940722	−1.6485558
Point Group = C1, Order = 1, Nsymop = 1.			

cis-flocoumafen (FCF): −1873.06139 au

	**Cartesian Coordinates (Angstroms)**		
**Atom**	**X**	**Y**	**Z**
1 H	−1.9833278	2.2425827	2.9916642
2 C	−1.9602973	2.0027236	1.9206003
3 C	−3.0398044	0.3883850	0.2367608
4 C	−2.5709891	3.2038753	1.1754090
5 C	−2.8876515	0.7826844	1.7216136
6 H	−2.0830530	−0.0405731	−0.0822910
7 H	−3.4342807	3.5521773	1.7598467
8 H	−3.8720804	1.0252961	2.1373436
9 H	−1.8617988	4.0396743	1.1585803
10 H	−2.5033272	−0.0796866	2.2765385
11 C	−0.5063744	1.6938820	1.5821978
12 C	2.1905838	1.0283509	1.0542865

13 C	0.2833264	1.0244431	2.5339457
14 C	0.0980922	2.0245780	0.3667560
15 C	1.4322233	1.7002136	0.0937481
16 C	1.6072103	0.6945716	2.2838335
17 H	−0.1509938	0.7600138	3.4955951
18 H	−0.4722434	2.5419472	−0.3970377
19 H	1.8563709	1.9765539	−0.8643062
20 H	2.2106992	0.1805328	3.0248678
21 O	3.5016666	0.6608455	0.9024518
22 C	4.1349301	0.9072200	−0.3414614
23 H	4.1759908	1.9875485	−0.5445351
24 H	3.5564502	0.4444853	−1.1561039
25 C	5.5329842	0.3328715	−0.3177460
26 C	8.1167470	−0.7502954	−0.3751208
27 C	5.9247627	−0.6117164	0.6336013
28 C	6.4509079	0.7315379	−1.2987561
29 C	7.7325591	0.1945015	−1.3325522
30 C	7.2114611	-1.1498457	0.6069695
31 H	5.2214826	-0.9175600	1.3987795
32 H	6.1621064	1.4716594	−2.0410242
33 H	8.4393024	0.5147720	−2.0912557
34 H	7.5120458	−1.8769249	1.3529945
35 C	9.4971286	−1.3411635	−0.4432598
36 F	9.6276213	−2.1888206	−1.4916283
37 F	10.4411105	−0.3838223	−0.6042990
38 F	9.8123969	−2.0361876	0.6707724
39 C	−5.3956087	−0.5687524	0.2840373
40 C	−4.0538767	−0.7327963	0.0829611
41 C	−3.5334744	−2.0500905	−0.2682061
42 O	−2.3711742	-2.3317243	−0.4544252
43 O	−4.4597015	−3.0890936	−0.4269068
44 O	−5.8520462	0.6589656	0.6310733
45 H	−6.8094758	0.7087707	0.5145573
46 C	−3.3063654	1.5916756	−0.6706547
47 C	−3.0410346	2.9033586	−0.2373060
48 C	−3.7415402	1.3909839	−1.9886677
49 H	−3.9302724	0.3780807	−2.3329765
50 C	−3.9329701	2.4563624	−2.8635328
51 H	−4.2700115	2.2701876	−3.8793253
52 C	−3.6804468	3.7590681	−2.4299946
53 H	−3.8190598	4.6010774	−3.1020472
54 C	−3.2334919	3.9691776	−1.1291263
55 H	−3.0235561	4.9806982	−0.7885773
56 C	−5.7900368	−2.9221669	−0.2177457
57 C	−8.5297392	−2.7020396	0.2328179
58 C	−6.6081120	−4.0431695	−0.3803270
59 C	−6.3260861	−1.6768773	0.1551861

60 C	−7.7111712	−1.5927475	0.3912802
61 C	−7.9745675	−3.9284706	−0.1580617
62 H	−6.1503606	−4.9814869	−0.6732736
63 H	−8.1589216	−0.6592326	0.7241146
64 H	−8.6126020	−4.7978164	−0.2826152
65 H	−9.5953943	−2.6190819	0.4185350
Point Group = C1, Order = 1, Nsymop = 1.			

Separation and purification of *cis* and *trans* forms of flocoumafen (1) via recrystallization

Even though the purity of isolated flocoumafen (**1**) was fully satisfactory with 99%, we need figure out structural conformation as *cis* and *trans* forms. Thus, we examined recystallization of **1** after preparation of **1** through coupling reaction, which was treated with appropriate solvent or its mixture such as ethyl acetate, acetone, diethyl ether, and hexane in order to provide structurally high purity of flocoumafen (**1**) (Table 2). We carried out the co-solvent system (Table 2, entries 4–8) showed superior purity and yield, whereas single solvent system (Table 2, entries 1–3) resulted relatively low purity and yields. Among many recrystallization trials, the ethyl acetate system showed the best result with purity and yield (Table 2, entries 1). In addition, an excellent result on the flash column chromatography under the standard condition using ethyl acetate/cyclohexane is shown in entry 7 of Table S3.

Table S3. Purification of flocoumafen (**1**).

**Entry**	**Solvent** [a]	**Purification Condition** [a]	**Purity** [b] **(%)**	**Ratio** [c] **(*cis*:*trans*)**	**Yield** [d] **(%)**
1	Ethyl acetate	0 °C, 2 h.	97.5	99:1	43
2	Acetone	0 °C, 2 h.	93.5	81:19	63
3	Diethyl ether	rt, 1 h.	92.8	52:48	10
4	Ethyl acetate/Hexane (9:1, v/v)	rt, 1 h.	96.7	81:19	12
5	Ethyl acetate/Diethyl ether (2:8, v/v)	0 °C to rt, 2 h.	89.4	45:45	30
6	Ethyl acetate/Hexane (1:2, v/v)	rt, (FCC) [e]	94.5	68:32	38
7	Ethyl acetate/Hexane (1:4, v/v)	rt, (FCC) [e]	99.9	58:42	35
8	Ethyl acetate/Hexane (1:4, v/v)	rt, (FCC) [e]	92.08	2:98	28

[a] Purified methods; entries 1-7: The crude FCF was dissolved to the solvents until clean solution and the mixture was evaporated excess solvent to reach half amounts and then the mixture was stirred at 0 °C or rt.; entries 1–7: The crude FCF was purified using flash column chromatograghy after recrystallization; [b] Purity was determinded based on analytical HPLC using analytical column: SC_{18}; symmetry C_{18} 5 μm, 3.9 × 150 mm, waters; [c] The ratio is structural isomer cis and trans; [d] Isolated pure yield; [e] FCC: Flash column chromatography.
